# Temporal Dynamics and Developmental Maturation of Salience, Default and Central-Executive Network Interactions Revealed by Variational Bayes Hidden Markov Modeling

**DOI:** 10.1371/journal.pcbi.1005138

**Published:** 2016-12-13

**Authors:** Srikanth Ryali, Kaustubh Supekar, Tianwen Chen, John Kochalka, Weidong Cai, Jonathan Nicholas, Aarthi Padmanabhan, Vinod Menon

**Affiliations:** 1 Department of Psychiatry and Behavioral Sciences, Stanford University, Stanford, United States of America; 2 Department of Neurology and Neurological Sciences, Stanford University, Stanford, United States of America; 3 Stanford Neurosciences Institute, Stanford University, Stanford, United States of America; Ghent University, BELGIUM

## Abstract

Little is currently known about dynamic brain networks involved in high-level cognition and their ontological basis. Here we develop a novel Variational Bayesian Hidden Markov Model (VB-HMM) to investigate dynamic temporal properties of interactions between salience (SN), default mode (DMN), and central executive (CEN) networks—three brain systems that play a critical role in human cognition. In contrast to conventional models, VB-HMM revealed multiple short-lived states characterized by rapid switching and transient connectivity between SN, CEN, and DMN. Furthermore, the three “static” networks occurred in a segregated state only intermittently. Findings were replicated in two adult cohorts from the Human Connectome Project. VB-HMM further revealed immature dynamic interactions between SN, CEN, and DMN in children, characterized by higher mean lifetimes in individual states, reduced switching probability between states and less differentiated connectivity across states. Our computational techniques provide new insights into human brain network dynamics and its maturation with development.

## Author Summary

Characterizing the temporal dynamics of functional interactions between distributed brain regions is of fundamental importance for understanding human brain organization and its development. Progress in the field has been hampered both by a lack of strong computational techniques to investigate brain dynamics and an inadequate focus on core brain systems involved in higher-order cognition. Here we address these gaps by developing a novel variational Bayesian Hidden Markov Model (VB-HMM) that uncovers non-stationary dynamical functional networks in human fMRI data. In two cohorts of adults, VB-HMM revealed multiple short-lived states characterized by rapid switching and transient connectivity between the salience (SN), default mode (DMN), and central executive (CEN) networks—three brain systems critical for higher-order cognition. In children, relative to adults, VB-HMM revealed immature dynamic interactions between SN, CEN, and DMN, characterized by higher mean lifetimes in individual states, reduced switching probability between states and less differentiated connectivity across states. Our findings suggest that the flexibility of switching between distinct brain states is weaker in childhood, and they provide a novel framework for modeling immature brain network organization in children. More generally, the approach used here may prove useful to the investigation of dynamic brain organization in neurodevelopmental and psychiatric disorders.

## Introduction

Our ability to adapt to a constantly changing environment is thought to depend on the dynamic and flexible organization of intrinsic brain networks [1,2]. Characterizing the temporal dynamics of interactions between distributed brain regions is fundamental to our understanding of human brain organization and its development [2–8]. However, most of our current knowledge of functional brain organization in adults and children is based on investigations of time-independent functional coupling. Progress in the field has been impeded by both a lack of appropriate computational techniques to investigate brain dynamics as well as an inadequate focus on core brain systems involved in higher-order cognition [3,4,9,10]. In particular, progress has been limited by weak analytical models for identifying time-varying brain states, and their occurrence rates and mean lifetimes, for quantifying transition probabilities between brain states, and for characterizing the dynamic evolution of functional connectivity patterns over time [9–11].

Here we overcome limitations of extant methods by developing and applying novel computational techniques for characterizing dynamic functional interactions between distributed brain regions and address two key neuroscientific goals. The first scientific goal of our study was to investigate the dynamic functional connectivity of the salience network (SN), the central-executive network (CEN) and the default mode network (DMN), three core neurocognitive systems that play a central role in cognitive and affective information processing [1,12]. Our second scientific goal was to characterize the maturation of the dynamic functional connectivity of the SN, CEN and DMN between childhood and adulthood in order to address important gaps in the literature regarding the nature of dynamic cross-network interactions over development and the question of how brain systems become more flexible during the period between childhood and adulthood.

The SN is a limbic-paralimbic network anchored in the anterior insula and dorsal anterior cingulate cortex with prominent subcortical nodes in affective and reward processing regions including the amygdala and ventral striatum [13,14]. The SN plays an important role in orienting attention to behaviorally and emotionally salient and rewarding stimuli and facilitating goal-directed behavior [12,14–16]. The fronto-parietal CEN is anchored in the dorsolateral prefrontal cortex and supramarginal gyrus and is critical for actively maintaining and manipulating information in working memory [17,18]. The DMN is anchored in the posterior cingulate cortex, medial prefrontal cortex, medial temporal lobe, and angular gyrus [19–21] and is involved in self-referential mental activity and autobiographical memory [22]. In adults, task-based fMRI studies have consistently demonstrated that SN, CEN and DMN nodes are involved in a wide range of cognitive tasks, with the strength of their responses increasing or decreasing proportionately with task demands [12,23,24]. Analysis of causal interactions between these networks has also shown that high-level attention and cognitive control processes rely on dynamic interactions between these three core neurocognitive networks [16,25–27]. Thus, far from operating independently, these three brain networks, which have only been probed using static time-invariant connectivity analysis, must form transient dynamic functional networks (DFNs) allowing for flexible within- and cross-network interactions.

While the SN, CEN and DMN can be reliably identified in most individuals using static network analysis of rs-fMRI data [26,28], progress in characterizing their dynamic temporal properties has been limited by currently available computational tools and procedures. Most current studies of dynamic brain connectivity use a sliding window approach [29,30], which is problematic because of arbitrary parameters such as window size, which can lead to erroneous estimates of dynamic connectivity [7,9,11]. Furthermore, extant methods do not provide information about the occurrence and lifetimes of individual dynamic brain states, transition probabilities between network states or unique dynamic network configurations associated with each brain connectivity state.

To address these weaknesses, we developed a novel variational Bayesian hidden Markov model (VB-HMM) [31] to uncover time-varying functional connectivity. HMM uses a state-space approach to model multivariate non-stationary time series data [32,33] and cluster them into distinct states, each with a different covariance matrix reflecting the functional connectivity between specific brain regions. Importantly, VB-HMM automatically prunes redundant states, retaining only those that significantly contribute to the underlying dynamics of the fMRI data, and provides the posterior distribution of parameters rather than point estimates of maximum likelihood-based methods.

We then used VB-HMM to characterize dynamic functional interactions between the SN, CEN and DMN to address our two neuroscientific goals. VB-HMM allowed us to examine for the first time several important metrics of brain dynamics: the number of distinct brain states, their occupancy rates and mean lifetimes, and switching probabilities between brain states and DFNs. Crucially, VB-HMM enabled us to investigate the temporal dynamics and evolution of states where the SN, DMN and CEN are fully segregated from each other, and states where they interact with each other. We hypothesized that segregation of the SN, DMN and CEN would constitute a dominant state with high occupancy rates and mean lifetimes. We further hypothesized that states with high occupancy rates would be temporally stable and marked by a higher probability of switching within the state compared to switching across states. We use sub-second resting-state fMRI (rs-fMRI) datasets acquired as part of the Human Connectome Project (HCP) (http://www.humanconnectome.org) and demonstrate the robustness of our findings across two independent cohorts of healthy adults.

Next, we used VB-HMM and insights from our analyses of the adult brain to characterize the maturation of dynamic functional networks and connectivity associated with the SN, DMN and CEN between childhood and adulthood. Flexible and dynamic cross-network functional interactions are essential for mature brain function [5,34], yet little is known about the nature of dynamic organization and time-varying connectivity in children relative to adults. Studies using static connectivity analyses suggest that functional brain networks undergo significant reconfiguration from childhood to adulthood, with analysis of time-averaged whole-brain connectivity patterns suggesting prominent increases as well as decreases in connectivity between childhood and adulthood. In a previous study we showed that time-averaged connectivity within key nodes of the SN and DMN as well as their inter-network interactions is weaker in children relative to adults [28]. Recent reports suggest that time-varying connectivity between distributed brain areas changes significantly with age, with greater temporal variability of connection strengths in children compared to adults[34]. Based on these observations, we hypothesized that compared to adults, children would show immature and less flexible patterns of dynamic connectivity between the SN, CEN and DMN. Crucially, VB-HMM allowed us to, for the first time, probe developmental changes in dynamic networks properties including the occurrence rates and mean lifetimes of distinct brain states, such as those in which the SN, CEN and DMN are fully segregated from each other with decreased switching probabilities.

## Materials and Methods

### Ethics statement

This study was approved by the Stanford University Institutional Review Board. Written informed consent was obtained from all the subjects.

### VB-HMM

We first describe a novel VB-HMM framework we developed for characterizing dynamic brain networks in human fMRI data. In the following sections, we represent matrices by using uppercase letters while scalars and vectors are represented using lowercase letters. Let $Y=\{\{y_{t}^{s}{\}}_{t=1}^{T}{\}}_{s=1}^{S}$ be the observed voxel time series, where *T* is the number of time samples and *S* is the number of subjects. $y_{t}^{s}$ is an *M* dimensional time sample at time *t* for subject *s*, where *M* is the number of brain regions or nodes of the dynamic functional network under investigation. Let $Z=\{\{z_{t}^{s}{\}}_{t=1}^{T}{\}}_{s=1}^{S}$ be the underlying hidden/latent discrete states, where $z_{t}^{s}$ is the state label at time *t* for subject *s*. Let *Z* be a first order Markov chain, with stationary transition (*A*) and initial distributions (*π*) defined as:

$$p(z_{t}^{s}=k|z_{t-1}^{s}=j)=A_{jk}$$ (1)

$$p(z_{1}^{s}=k)=\pi_{k}$$ (2)

where $0\leq A_{jk}\leq 1,\sum_{k=1}^{K}A_{jk}=1$, and $\pi_{k}\geq 0,\;\sum_{k=1}^{K}\pi_{k}=1$.

We assume the probability of the observation $y_{t}^{s}$ given its state $z_{t}^{s}=k$ to be a multivariate normal distribution with parameters mean *μ*_*k*_ and covariance Σ_*k*_:

$$p(y_{t}^{s}|z_{t}^{s}=k)=N(\mu_{k},\Sigma_{k})$$ (3)

Here we assume that the number of possible states *K* is not known a priori. Each state *k* has *M μ*_*k*_ and an *M* x *M* Σ_*k*_.

**Fig 1:**
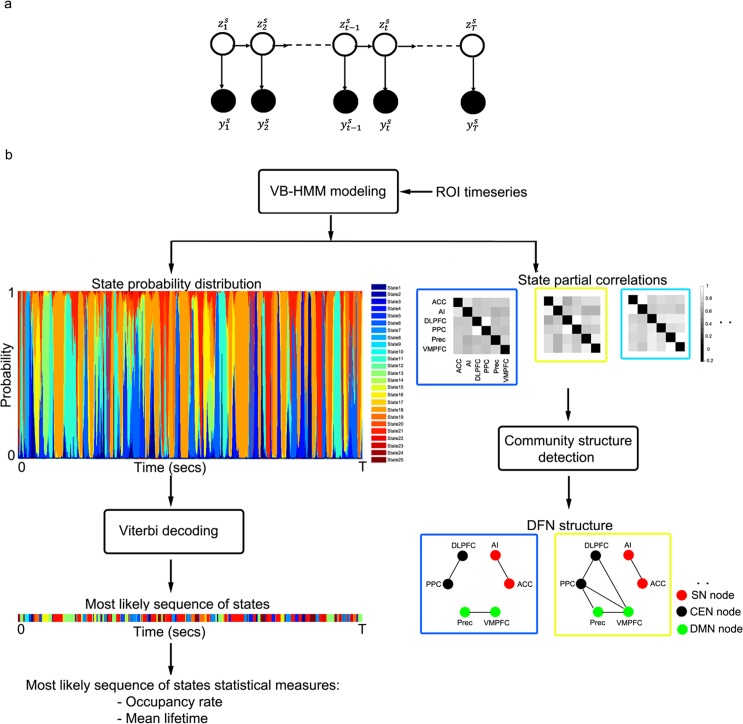
**(a) Generative model for Hidden Markov Model (HMM).** HMM is a state-space model consisting of latent discrete variables $z_{t}^{s}$ and observed rs-fMRI time series $y_{t}^{s}$ for each subject *S*. The discrete variables $z_{t}^{s}$ form a Markov chain with transition probabilities given by a multinomial distribution *A*_*i*,*j*_. For a given state *k*, we model the rs-fMRI time series as a multi-normal distribution *N*(*μ*_*k*_,*C*_*k*_), with mean *μ*_*k*_ and covariance *C*_*k*_. Given rs-fMRI datasets for S subjects, we estimate the posterior probabilities of the latent states and model parameters Φ = {*π*,*A*,Θ} (where, ${\Theta =\{\mu }_{k},\Sigma_{k}{\}}_{k=1}^{K}$) using variational Bayes methods. **(b) Flowchart for VB-HMM.** (i) Extract time series from *M* ROIs of *S* subjects; (ii) Use VB-HMM to estimate the posterior probabilities of states and model parameters Φ = {*π*,*A*,Θ}; (iii) Apply Viterbi decoding to estimate the most probable states; (iv) Compute the occupancy rate and mean lifetime of each state; and (v) Apply Louvain community detection on the estimated partial correlations to find network communities within each state. In the left panel, each of the 25 colors depicts a different state. The colors in the left panel match the colors used in the partial correlation matrices on the right.

Let Φ = {*π*,*A*,Θ} (where ${\Theta =\{\mu }_{k},\Sigma_{k}{\}}_{k=1}^{K}$) be the unknown parameters of the HMM model. Using the factorization property [35] of the Bayesian network shown in **Fig 1A**, the joint probability distribution of the observations, hidden states, and parameters can be written as

$$p(Y,Z,\Phi )=\prod_{s=1}^{S}p(z_{1}^{s}|\pi )\prod_{t=2}^{T_{s}}p(z_{t}^{s}|z_{t-1}^{s},A)\prod_{t=1}^{T_{s}}p((y_{t}^{s}|z_{t}^{s},\Theta )P(\Phi )$$ (4)

In maximum likelihood methods, the parameters Φ of the model are assumed to be unknown deterministic quantities, whereas in the Bayesian approach they are treated as random variables with prior probability distributions. Here we assume that conjugate priors [35] for Φ and *Z* are defined as in [31] with the goal of estimating the joint posterior distribution *p*(*Z*,Φ|Y) of the hidden states and parameters. Estimating this posterior distribution is analytically intractable but inference methods, such as sampling or variational methods, can instead be used [31,35]. Here, to estimate *p*(*Z*,Φ|Y), we use a variational Bayesian (VB) method [31], which not only provides an elegant analytical approximation to the required posterior distribution but is also computationally faster than sampling approaches.

Let *q*(Z,Φ|Y) be any arbitrary probability distribution and *p*(Z,Φ|Y) be the true posterior probability distribution. Then the log of the marginal distribution of observations Y can be written as

log⁡P(Y)=F(q)+KL(q||p) (5)

where *F*(*q*) is known as the negative free energy and *KL*(*q*||*p*) is the Kullback-Leibler (KL) divergence between the approximate and true posterior. These quantities are given by

$$F(q)=\int dZd\Phi q(Z,\Phi |Y)\;\log\frac{p(Y,Z,\Phi )}{q(Z,\Phi |Y)}$$ (6)

$$KL(q||p)=-\int dZd\Phi q(Z,\Phi |Y)\;\log\frac{p(Z,\Phi |Y)}{q(Z,\Phi |Y)}$$ (7)

Since *KL*(*q*||*p*) is nonnegative, *F*(*q*) serves as the strict lower bound on log *P*(*Y*). *F*(*q*) and log *P*(*Y*) are equal if and only if the approximate posterior *q*(*Z*,Φ|Y) is equal to the true posterior *p*(*Z*,Φ|Y) for which *KL*(*q*||*p*) = 0. The goal of VB approximation is to find the approximate posterior for which the lower bound *F*(*q*) is maximized. We make a mean field approximation on the approximate posterior [31] wherein it factorizes as

q(Z,Φ|Y)=q(Z,A,Θ,π|Y)=q(Z|Y)q(π|Y)q(A|Y)q(Θ|Y) (8)

The functional forms of these factors are defined by the priors on the parameters and the likelihood of the data. We assume conjugate priors for the priors, which results in elegant analytical approximations to the required posterior distributions of the Eq (8). Accordingly, the conjugate prior for *π* and rows of *A* is the Dirichlet (*Dir*) distribution, while the prior over the parameters of the Gaussian distribution Θ is the Normal-Wishart (*NW*) distribution. We further assume that the prior distribution over Φ factorizes as

P(Φ)=p(π)p(A)p(Θ) (9)

The forms of the Dirichlet and Normal-Wishart distributions are defined in [31]. We provide the values of the hyper-parameters of these distributions in the Appendix.

Since we define conjugate priors on the model parameters, *q*(*π*|*Y*) and *q*(*A*|*Y*) follow multinomial distributions and *q*(Θ|Y) follows the Normal-Wishart distribution [31]. The update equations for the posterior parameters are provided in the Supplementary Material. The posterior distribution of the hidden states can be estimated using an efficient forward-backward method similar to the Baum-Welch algorithm for ML-HMM [33,35]. Furthermore, our VB-HMM estimates the parameters of Normal-Wishart distribution for each state. VB-HMM therefore discovers states for which the parameters of the Normal-Wishart distributions are distinct for each state. A new state will be discovered if either mean or covariance or both are different in that state with respect to other states. In task-based fMRI studies it is important to discover states with both mean and covariance differences. However, in resting-state fMRI studies, as in the current study, differences in absolute signal levels are not relevant and states are based solely on changes in covariance over time. This can be accomplished elegantly in our Bayesian framework using the hyperparameter *λ*_*k*_ in the joint Normal-Wishart distribution. A non-informative prior value (*say*, *λ*_*k*_ = 0.001) allows the data to determine the joint posterior distributions for the mean and covariance. However, setting it to a very high value (*λ*_*k*_ = 1000) biases the posterior to the prior mean which is 0 in our case (equation S.10). This ensures that our states are discovered only by the changes in covariance/inverse covariance in each state.

Similar to the expectation maximization algorithm for ML-HMM, the posterior distributions for the latent and model parameters are iteratively updated in VB-HMM as follows:

1. *Initialize the latent variable* *Z*
2. *Update q(Θ|Y) given q(Z|Y)* *(M-step)*
3. *Update q(Z|Y) given q(Θ|Y)* *(E-step)*
4. *Iterate steps (b) and* *(c) until convergence*

We iterate steps (b) and (c) until the fractional lower bound *F*(*q*) between two consecutive thresholds is below a set threshold value of *tol* = 10^−3^. We initialize the states using the K-means algorithm with the number of clusters/states K set to a high value (K = 25). The sparsity property of VB-HMM prunes away unwanted clusters/states in the model. Like ML-HMM, VB-HMM provides suboptimal estimates of the posterior distributions, and these estimates are sensitive to the initial estimates of states using K-means initialization. To account for this, we repeat VB-HMM with 100 different random initializations and choose the solution for which the lower bound *F*(*q*) is maximum.

### Dynamic network properties

#### Occupancy rate and mean lifetime of each state

We computed the mean lifetime and occupancy of each state based on the posterior distribution of each state *q*(*Z*|*Y*) estimated by VB-HMM. We first applied the Viterbi algorithm [35] to estimate the most probable sequence of states z*. The occupancy of each state *k* is the amount of time spent in the state, which is computed by counting the number of time points in which a given state *k* occurs:

$$Occupancy\;rate\;(k)=\frac{\sum_{t=1}^{ST_{s}}I(z^{*}(t)=k)}{ST_{s}}*100$$ (10)

where *I*(z*(*t*) = *k*) is a Kronecker delta, which is 1 if the current state z*(*t*) is *k*. The mean lifetime of a state *k* is the average time for which that particular state is continuously present and is computed by counting how long that state continuously persists and then taking an average of those counts. We calculated both the occupancy rate and the mean lifetime of each state at the group level as well as at the single subject level.

#### State transition probability

The posterior distribution of state transition probability matrix *q*(*A*|*Y*) is estimated as part of the M step of VB-HMM. Note that VB-HMM estimates posterior probabilities *q*(*Z*^*s*^ = *k*|*Y*) of each state *k* and subject *s* while estimating the posterior distribution of model parameters *q*(*Θ*|*Y*) (including *q*(*A*|*Y*)), which are the same for the given set of subjects. Therefore, to estimate subject-specific state transition probabilities, we can take the following frequentist approach:

1. *Apply the Viterbi algorithm to estimate the most-likely discrete states for each subject q*(*Z*^*s*^ *= k|Y*).
2. *For each subject*, *let* (*C*^*s*^(*i*,*j*)*) be the number of times the state ′i′ and ′j′ occur consecutively at time ′t − 1′ and ′t′ (1 ≤ t ≤ T and 2 ≤ t − 1 ≤ T and every state 1 ≤ i*,*j ≤ K)*.
3. *For each subject s*, *let C*^*s*^(*i*) *be the number of times that state i occurs in that subject*.
4. *Compute the joint probability of states i and j for subject s as*
  $$p^{s}(z^{*}(t-1)=i,z^{*}(t)=j)=\frac{C^{s}(i,j)}{C^{s}(i)C^{s}(j)}$$ (11)
  *and the marginal probability of state i as*
  $$p^{s}(z^{*}(t)=i)=\frac{C^{s}(i)}{T}$$ (12)
  *The state transition probability A*^*s*^(*i*,*j*) *for subject s is then given by*
  $$A^{s}(i,j)=\frac{p^{s}(z^{*}(t-1)=i,z^{*}(t)=j)}{p^{s}(z^{*}(t)=i)}$$ (13)

#### Identification of time-varying networks

We first computed the partial correlations from the estimated covariance matrices for each state and then determined the dynamic functional community structure under each state by applying the Louvain community detection algorithm [36] on the estimated partial correlations. A Louvain algorithm that takes into consideration positive and negative relationships/values was applied to the unthresholded partial correlation matrix. Negative values were treated asymmetrically. Specifically, we used the Matlab function *community_louvain* made available in the widely-used Brain Connectivity ToolBox (www.brain-connectivity-toolbox.net). Network representation of states provides a level of abstraction that is easy to interpret and more importantly allows us to relate our findings to extant literature that mostly describes intrinsic functional connectivity findings in terms of within- and between- network coupling.

Note that in our Bayesian framework, for each state k, the posterior distribution for mean *μ*_*k*_ and covariance Σ_*k*_ is a Normal-Wishart distribution. We estimate the parameters for this joint posterior distribution in each state. More specifically, the Wishart distribution gives the probability distribution over the inverse covariance (precision) matrix ($\Sigma_{k}^{-1}$) for each state. The expected value of the covariance matrix for each state k is given by $b_{k}^{\prime }/a_{k}^{\prime }$ (Eqs S.11 and S.13). More importantly, the covariance matrix estimation in our Bayesian framework is regularized by the prior parameters and the degree of regularization is determined automatically from the data (Eqs S.10–S.15). This regularization ensures that the covariance (or inverse covariance) matrix is full rank. The required partial correlations for each state k are then computed by properly scaling the estimated inverse covariance matrix.

### Validation datasets

We validated VB-HMM using three different simulation models; the details of each are provided in the Supplementary Materials. Briefly, in *Simulation-1*, we created datasets with two nodes and two hidden states. The hidden states were constructed using a typical block design with two conditions (or states): “OFF” and “ON” as shown in **S2A Fig**. We simulated observations with two nodes where the nodes are negatively correlated in the “OFF” state and uncorrelated in the “ON” state. In *Simulation-*2, we simulated data with six nodes and two hidden states using the HMM generative model given by Eqs 1–3. In this case, the two hidden states were constructed using a specified state transition matrix *A* and six nodes/ROIs with observations drawn from a zero-mean multivariate Gaussian distribution and state specific covariance matrices (**S3A Fig**). *Simulation-3* also consisted of six nodes and two hidden states. Here, however, the first three nodes/ROIs were correlated in the first half (116 samples) of the experiment (state 1) while the other three ROIs were correlated in the second half (116 samples) of the experiment (state 2) (**S4A Fig**). Five datasets were simulated (akin to a group size of five subjects in fMRI studies) for each simulation type.

### HCP rs-fMRI datasets

#### Data description

Minimally preprocessed rs-fMRI data were obtained from the HCP (http://www.humanconnectomeproject.org) under the Q1-Q6 Data Release. Forty individuals (first session, left-right encoded, ages: 22–35, 28 males) were selected from 500 individuals based on the following criteria: (1) individuals were unrelated; (2) range of head motion in any translational direction was less than 1 mm; (3) average scan-to-scan head motion was less than 0.2 mm; and (4) maximum scan-to-scan head motion was less than 1 mm. For each individual, 1,200 frames were acquired using multiband, gradient-echo planar imaging with the following parameters: TR, 720 ms; echo time, 33.1 ms; flip angle, 52°; field of view, 280 × 180 mm; matrix, 140 × 90; and voxel dimensions, 2 mm isotropic. During scanning, participants kept their eyes open and fixated on a crosshair on the center of the screen. We divided the group into two cohorts of 20 participants each (hereafter referred to as Cohorts 1 and 2); the two cohorts did not differ on age, gender, IQ, or other demographic measures (*p* > 0.5).

#### Data pre-processing

Spatial smoothing with a Gaussian kernel of 6mm FWHM was first applied to the minimally preprocessed data [37] to improve the signal to noise ratio as well as the anatomy correspondence between individuals. A multiple linear regression approach with 12 realignment parameters (3 translations, 3 rotations, and their first temporal derivatives) was applied to the smoothed data to reduce head motion-related artifacts. Additionally, low frequency drifts were removed.

#### Regions of Interest (ROIs)

Key nodes of the triple network model, involving the SN, CEN, and DMN, were determined based on a model of their differential roles in saliency detection and cognitive control [38,39]. Meta-analyses of cognitive control tasks have consistently reported right-hemisphere dominant activation, most notably in the right anterior insula [40–42]. Accordingly, our analysis focused on right anterior insula and anterior cingulate nodes of the SN, right dorsolateral and supramarginal gyrus nodes of the CEN, and the precuneus and ventromedial prefrontal cortex nodes of the DMN, similar to previous studies [12,26,43,44]. The six ROIs were determined in an unbiased manner using an independent dataset and a previously published study [45]; the ICA maps are shared through brainmap.org and FMRIB’s website (http://fsl.fmrib.ox.ac.uk/analysis/brainmap+rsns/). Crucially, these procedures allowed us to characterize network dynamics in the context of canonical static networks identified by ICA.

From the SN map, we identified peak activations in the right anterior insula (AI, MNI coordinates: x = 36; y = 22; z = 8) and anterior cingulate cortex (ACC, MNI coordinates: x = 2; y = 12; z = 44). From the ICA map of the CEN, we identified peaks in the right dorsolateral prefrontal cortex (DLPFC, MNI coordinates: x = 34; y = 18; z = 60) and posterior parietal cortex (PPC, MNI coordinates: x = 44; y = -54; z = 58). From the ICA map of the DMN, we identified peaks in the precuneus (Prec, MNI coordinates: x = -10; y = -62; z = 20) and ventromedial prefrontal cortex (VMPFC, MNI coordinates: x = 4; y = 54; z = -8). ROIs were constructed using spheres with centers as the local peaks and a radius of 6 mm. **S1 Fig** shows the anatomical locations of these nodes.

For each individual, the first eigenvariate of the voxel time series from each ROI was extracted. The first 8 frames were discarded to minimize the non-equilibrium effects in fMRI signal. The resulting time series were further high-pass filtered (*f* > 0.008 Hz) to remove low-frequency signals related to scanner drift.

#### VB-HMM analysis

We applied VB-HMM on rs-fMRI data from the first 20 participants (Cohort 1) and then conducted a replication analysis on rs-fMRI data from the second independent group of 20 participants (Cohort 2). VB-HMM finds an optimal model while pruning away redundant states. The estimated model consists of (a) switching probabilities between states for each subject in the cohort and (b) estimated covariance matrices for each state. We then apply the following steps on the estimated model to infer the underlying dynamic network structures in the rs-fMRI data.

1. *Apply the Viterbi algorithm to find an optimal sequence of the discrete states from the switching probabilities*.
2. *Compute the mean and standard deviations of occupancy rate and mean lifetime of each state across all subjects*.
3. *Compute partial correlations from the estimated covariance matrices for each state*.
4. *Apply modularity-based community detection on the partial correlations of each state*.
5. *Merge states having the same community structure into one state*.
6. *Recompute the mean and standard deviations of occupancy rate and mean lifetime of the merged states across all subjects*.

**Fig 1B** shows the flow chart of the various steps involved in inferring dynamic functional networks using VB-HMM.

#### Community detection and state merging

Though VB-HMM identifies different states based on differences in the covariance/partial correlations between brain regions, the underlying community structures for the estimated partial correlations can be the same. Since we are interested in identifying the functional community structure for each state, we merge those states that have the same functional community structure into one state.

### Stanford rs-fMRI data from children and adults

#### Data description

To investigate developmental changes in dynamic functional interactions across the three networks between childhood and adulthood, we used rs-fMRI data from twenty-four healthy children (12 males, 12 females, ages 7–9) and twenty-four healthy young adults (12 males, 12 females, age: 19–22) [46]. Participant demographics and statistics, including age, IQ and gender, are summarized in **S1 Table**. Children and adults were recruited from the San Francisco Bay Area as part of ongoing studies of brain and cognitive development. All participants were right-handed, had no history of neurological or psychiatric diseases, and were not currently taking any medication. All participants had an intelligence quotient (IQ) between 95 and 135 as measured by the Wechsler Abbreviated Scales of Intelligence (WASI). The study protocol was approved by the Stanford University Institutional Review Board. Written informed consent was obtained from each participant as well as the child's legal guardian before participation.

#### fMRI data acquisition

For each individual, 240 frames were acquired from a 3T GE Signa scanner (General Electric) using a custom-built head coil with a T2*-sensitive gradient echo spiral in-out pulse sequence based on blood oxygenation level-dependent (BOLD) contrast. Twenty-nine axial slices (4.0 mm thickness, 0.5 mm skip) parallel to the anterior and posterior commissure (AC-PC) line and covering the whole brain were imaged with the following parameters: volume repetition time (TR) = 2.0 s, echo time (TE) = 25 ms, 80° flip angle, matrix size 64 × 64, field of view 200 × 200 mm, and an in-plane spatial resolution of 3.125 mm. To reduce blurring and signal loss arising from field inhomogeneities, an automated high-order shimming method based on spiral acquisitions was used before acquiring functional images. A linear shim correction was applied separately for each slice during reconstruction using a magnetic field map acquired automatically by the pulse sequence at the beginning of the scan. Children and adults did not differ on movement (**S1 Table**).

#### Data preprocessing

Images were preprocessed using Statistical Parametric Mapping (SPM8, http://www.fil.ion.ucl.ac.uk/spm). The first eight volumes were discarded for stabilization of the MR signal. The remaining functional images were realigned to correct for rigid body motion. Subsequently, images were slice timing corrected, normalized into a standard stereotactic space, and resampled into 2 mm isotropic voxels. Finally, images were spatially smoothed by convolving an isotropic 3D Gaussian kernel (6 mm full width at half maximum).

#### VB-HMM analysis

ROIs and analysis procedures were the same as above.

## Results

### VB-HMM analysis of simulated datasets

We first validated VB-HMM using computer-simulated datasets generated from three different simulation models. Here we briefly summarize the results from these simulations; details are in the Supplementary Materials. For all three simulations, we applied VB-HMM with the number of hidden states (K) initialized to 25 and used VB-HMM to automatically determine the optimal number of states from the data. **S2 Fig** shows the actual states, the estimated posterior probabilities and the Viterbi decoded states for *Simulation-1*. Among the 25 states, the occupancy rates of 18 states are zero suggesting that VB-HMM penalizes redundant states. Further analysis suggests that among the seven with non-zero occupancy rates, four states together constitute 98% of the total occupancy rate and these states match the underlying true states in terms of their associated estimated Pearson correlation matrices and their occurrences with respect to their respective true states. Similarly, 21 out of the 25 states in *Simulation-2* had zero occupancy rates (**S3 Fig**). The top two most dominant states comprise 98% of the total occupancy rate and are well matched with the temporal occurrence of the underlying actual states. Lastly, *Simulation-3* yielded 21 out of 25 states with an occupancy rate of zero (**S4 Fig**). Of the four states with non-zero occupancy rates, the top two account for 99.2% of the total occupancy rate and match the true states used to generate the data. These simulations demonstrate that VB-HMM can accurately discover the optimal number of states and the underlying dynamic connectivity across different models of simulated data.

### Dynamic SN, DMN and CEN connectivity in multi-cohort HCP data

We applied VB-HMM on rs-fMRI data to uncover dynamic functional interactions between the SN, CEN and DMN in two cohorts of HCP data. Our first goal here was to identify dynamic brain states and their associated functional networks. We computed the occupancy rates and mean lifetimes of each state as well as the switching probabilities between states. A particular theoretical focus was on the occurrence of brain states in which the three networks were disconnected from each other. We conducted separate analyses on Cohorts 1 and 2 and investigated the robustness and consistency of our key findings across the two cohorts.

### Dynamic brain states, occurrence rates and mean lifetimes

#### HCP Cohort 1

**Fig 2:**
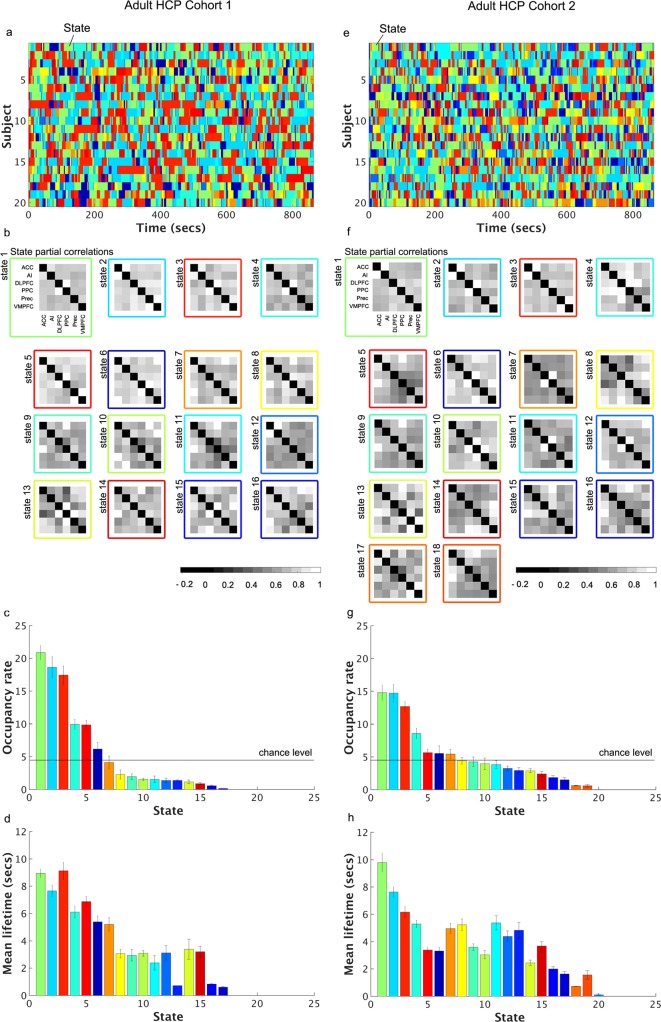
Dynamic states discovered by VB-HMM in two adult HCP cohorts. **Adult HCP Cohort 1: (a)** Time evolution of dynamic states in each subject. VB-HMM uncovers states at the group level thereby eliminating the matching of states across subjects. The state with the highest occupancy rate was assigned the first color in the jet colormap, the state with the second highest occupancy rate was assigned the second color in the jet colormap, and so on.; **(b)** Estimated partial correlations; **(c)** Occupancy rate. The occupancy of the top 5 states was significantly different from chance-level occupancy rate (data plotted; see SI for details); and **(d)** Mean lifetimes of each state. The mean lifetime of all states was significantly different from chance-level mean lifetime (data not plotted; see SI for details). **Adult HCP Cohort 2: (e)** Time evolution of dynamic states in each subject. The states coloring procedure was same as Adult HCP Cohort 1; **(f)** Estimated partial correlations; **(g)** Occupancy rate. The occupancy of the top 5 states was significantly different from chance-level occupancy rate (data plotted; see SI for details); and **(h)** Mean lifetimes of each state. Among the 25 initial states, only 16 states and 19 states have non-zero occupancy rates in Adult HCP Cohorts 1 and 2, respectively. In each cohort, only the first three states have occupancy rates above 10%. These high-occupancy states have mean lifetimes of about 7-10s, suggesting that the temporal persistence of these states is much shorter than the length of the scan.

VB-HMM identified 16 dynamic functional states, their time evolution, and the functional connectivity between nodes of the SN, CEN and DMN in each state (**Fig 2A and 2B**). All 16 functional states had non-zero occupancy rates and among them only three had rates above 10% (**Fig 2C**). The mean lifetime of the top three high-occupancy states was 7–9 s (**Fig 2D**), indicating temporal persistence over durations much shorter than the length of the scan (864 s). The occupancy rate and mean lifetime of states were not significantly correlated with age, gender, and mean framewise displacement (*q* > 0.05). Additionally, we did not find a state or a subset of states that was consistently related to high or low motion periods across subjects (*q* > 0.05).

#### HCP Cohort 2

We repeated the same analyses using data from HCP Cohort 2. VB-HMM identified 19 states with non-zero occupancy rates, of which, again, only three had occupancy rates above 10% (**Fig 2E–2G**). As with Cohort 1, the mean lifetime of the top three states was 6–10 s (**Fig 2H**), confirming temporal persistence over durations much shorter than the length of the scan. Similar to cohort 1, the occupancy rate and mean lifetime of states were not significantly correlated with age, gender, and mean framewise displacement (*q* > 0.05). Additionally, we did not find a state or a subset of states that was consistently related to high or low motion periods across subjects (*q* > 0.05).

### Occurrence rates, mean lifetimes, and connectivity in dynamic functional networks (DFNs)

To characterize the connectivity patterns associated with each functional state, we used a community detection algorithm on the estimated partial correlations in each state and examined the functional connectivity between ROIs. Below we describe the salient features of the dynamic functional network structure in each cohort. Given our focus on the temporal properties of the state in which the SN, CEN, and DMN were disconnected from each other, we combined states with a similar community structure into distinct DFNs (see **S1 Text**). We then examined the occupancy rates, mean lifetimes and switching probabilities of these DFNs.

#### HCP Cohort 1

**Fig 3:**
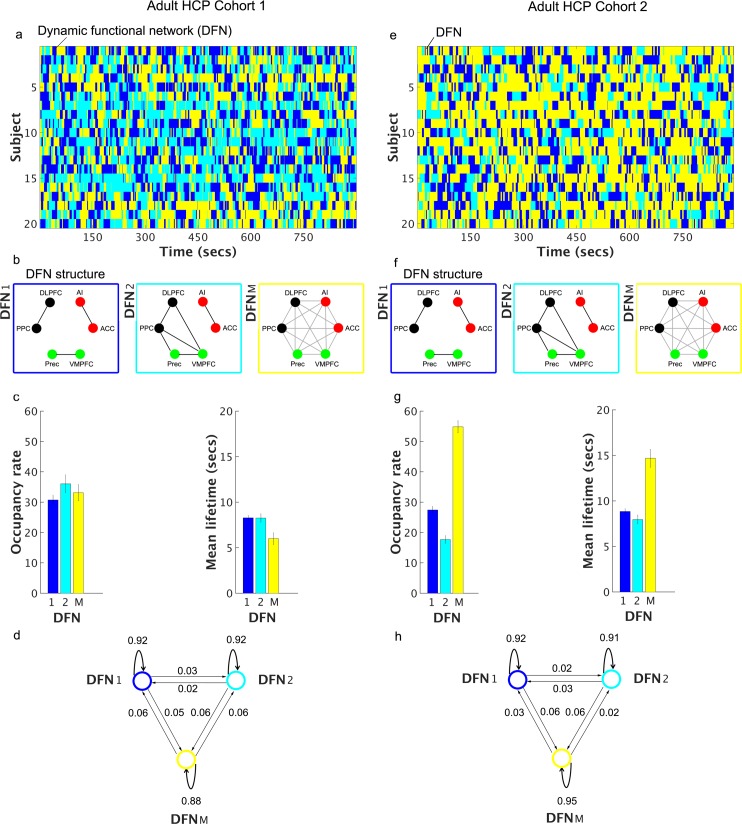
Dynamic functional networks identified in two adult HCP cohorts. **Adult HCP Cohort 1: (a)** Time evolution of merged dynamic networks in each subject; **(b)** Dynamic functional networks (DFNs) for the merged states 1 and 2 and the mixed state; **(c)** Occupancy rates and mean lifetimes of the DFNs; and **(d)** Switching probabilities between the DFNs. **Adult HCP Cohort 2: (e)** Time evolution of merged dynamic networks in each subject; **(f)** Dynamic functional networks (DFNs) for the merged states 1 and 2 and the mixed state; **(g)** Occupancy rates and mean lifetimes of the DFNs; and **(h)** Switching probabilities between the DFNs. In both cohorts, the most dominant DFN (DFN-1) consists of nodes in the SN, CEN, and DMN, which constitute three independent communities. DFN-1 has an occupancy rate of 31% (SEM: ± 1.58%) with a mean lifetime of 8.3s (SEM: ± 0.29s) for Adult HCP Cohort 1 and an occupancy rate of 27% (SEM: ± 1.2%) with a mean lifetime of 8.8s (SEM: ± 0.31s) for Adult HCP Cohort 2. In the second most dominant DFN (DFN-2) in both cohorts, the nodes of the SN form an independent community, while the nodes of the CEN and DMN interact and form one community. DFN-2 has an occupancy rate of 36% (SEM: ± 2.9%) with a mean lifetime of 8.2s (SEM: ± 0.47s) in Adult HCP Cohort 1 and an occupancy rate of 18% (SEM: ± 1.27%) with a mean lifetime of 8.0s (SEM: ± 0.48s) in Adult HCP Cohort 2. In both cohorts, the self-transition probabilities from a DFN to itself are high (0.85 ± 0.01 for Adult HCP Cohort 1 and 0.9 ± 0.05 for Adult HCP Cohort 2), while the transitions between DFNs are low (0.08 ± 0.01 for Adult HCP Cohort 1 and 0.06 ± 0.02 for Adult HCP Cohort 2).

We identified two DFNs with distinct community structures (**Fig 3A and 3B**). In DFN-1, the SN, CEN and DMN were disconnected from each other and formed three independent communities (States 1 and 5 in **S5 Fig**). Notably, this disconnected network configuration had an occupancy rate of 31% ± 1.58% and a mean lifetime of 8.3s ± 0.29s (**Fig 3C**). The network configuration that showed the next highest occupancy rate was one in which the CEN and DMN were connected with each other while the SN formed an independent community (States 2 and 3 in **S5 Fig**). This connected network configuration (DFN-2 in **Fig 3B**) had an occupancy rate of 36% ± 2.9% with a mean lifetime of 8.2s ± 0.47s (**Fig 3C**). All other network configurations had occurrence rates of 10% or less (**S5 Fig**). We then compared DFN-1 and DFN-2 connectivity profiles by examining two different types of links: cross-network, in which links spanned different static networks (SN, CEN, and DMN), and within-network, in which links did not span across nodes of the three static networks. We found a significant two-way interaction (*F*_*1*,*19*_ = 4.943, *p* = 0.039, **S7A Fig**), such that the connectivity of cross-network links was greater in DFN-2 compared to DFN-1 (*p* < 0.001) while no significant difference was observed between DFN-1 and DFN-2 for within-network links (*p* = 0.461). These results demonstrate that the two DFNs differ significantly in their connectivity profiles.

#### HCP Cohort 2

We repeated the same analysis using data from Cohort 2. We identified two DFNs with distinct community structures (**Fig 3E and 3F**). In DFN-1, the SN, CEN and DMN were disconnected from each other and formed three independent communities (States 2 and 3 in **S6 Fig**). This disconnected network configuration (DFN-1 in **Fig 3F**) had an occupancy rate of 27% ± 1.2% (**Fig 3G**) and a mean lifetime of 8.8s ± 0.31s (**Fig 3G**). The network configuration that showed the next highest occupancy rate was one in which the CEN and DMN were connected with each other, while the SN formed an independent community (States 1 and 14 in **S6 Fig**). This connected network configuration (DFN-2 in **Fig 3F**) had an occupancy rate of 18% ± 1.27% with a mean lifetime of 8.0s ± 0.48s (**Fig 3G**). All other network configurations had occurrence rates of 10% or less (**S6 Fig**). As in Cohort 1, we then compared DFN-1 and DFN-2 connectivity profiles by examining two different types of links: cross-network and within-network as described in the previous section. We found a significant interaction between DFN and link type (*F*_*1*,*19*_ = 40.87, *p* < 0.001, **S7B Fig**) such that the strength of cross-network links was greater in DFN-2 compared to DFN-1 (*p* < 0.001), while the reverse was true for within-network links (*p* < 0.001). These results confirm different connectivity profiles across the two DFNs.

In spite of differences in the occupancy rate and mean lifetimes of the mixed DFN-M across the cohorts (**Fig 3C and 3G)**, a noteworthy feature of the results is that in both Cohorts 1 and 2, DFN-1 and DFN-2 have the same underlying community structure, occupancy rates of 15–35% (**Fig 3C and 3G**), and the mean lifetimes of about 8 seconds (**Fig 3C and 3G**).

### Transitions between dynamic functional networks

Based on our primary goal of characterizing the network structure associated with segregated SN, CEN and DMN as encapsulated by DFN-1 (**Fig 3B**) and the common patterns of network structure involving DFN-1 and DFN-2 in both cohorts (see previous sections), we next examined state transitions between these networks. In each cohort, network structures corresponding to all other functional states were combined together into a mixed DFN-M. As in previous sections, these analyses were conducted separately in the two cohorts with the aim of elucidating replicable findings.

#### HCP Cohort 1

**Fig 3D** shows the average transition probabilities within and between the three network configurations: DFN-1, DFN-2, and DFN-M. We found that the self-transition probabilities from a DFN to itself were high (0.85 ± 0.01) while transitions between DFNs were low (0.08 ± 0.01). The high self-transition probabilities could be attributed to the Markovian aspect of the model and the temporal autocorrelations in fMRI timeseries data [47].

#### HCP Cohort 2

We repeated the above analysis on data from Cohort 2. **Fig 3H** shows the transition probabilities between DFN-1, DFN-2, and DFN-M. As in the case of Cohort 1, self-transitions from a network to itself were greater (0.9 ± 0.05) than transitions across networks (0.06 ± 0.02).

These findings suggest that DFN-1 and DFN-2 are relatively stable over time and that transitions within the same DFNs are much more likely than transitions between DFNs.

### Maturation of dynamic SN, DMN, and CEN connectivity in Stanford developmental data

We next used VB-HMM to characterize the maturation of dynamic functional interactions between the SN, CEN and DMN in a Stanford cohort of IQ- and gender-matched adults and children. We used the same analytic procedures as described above on data from adults and children and then compared dynamic network properties between the two groups.

### Occurrence and lifetime of dynamic brain states

#### Adults

**Fig 4:**
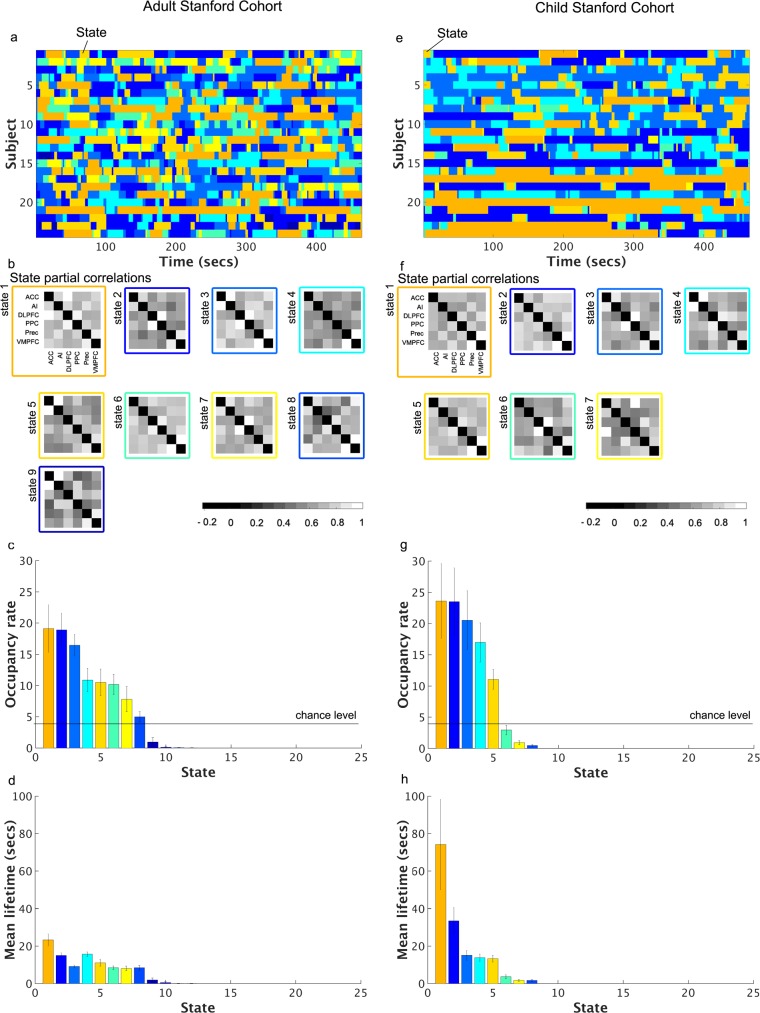
Dynamic states discovered by VB-HMM in Stanford developmental data. **Adult Stanford Cohort: (a)** Time evolution of dynamic states in each subject. VB-HMM uncovers states at the group level thereby eliminating the matching of states across subjects. The state with the highest occupancy rate was assigned the first color in the jet colormap, the state with the second highest occupancy rate was assigned the second color in the jet colormap, and so on; **(b)** Estimated partial correlations; (**c**) Occupancy rate of each state. The occupancy rate of the top 7 states was significantly different from chance-level occupancy rate (data plotted; see SI for details); and **(d)** Mean lifetimes of each state. The mean lifetime of all states was significantly different from chance-level mean lifetime (data not plotted; see SI for details). **Child Stanford Cohort: (e)** Time evolution of dynamic states in each subject; (**f**) Estimated partial correlations; (**g**) Occupancy rate of each state. The occupancy rate of the top 5 states was significantly different from chance-level occupancy rate (data plotted; see SI for details); and **(h)** Mean lifetimes of each state. The mean lifetime of all states was significantly different from chance-level mean lifetime (data not plotted; see SI for details) The states coloring procedure was same as the Adult Stanford Cohort. Among the 25 initial states, only nine states in the Adult Stanford Cohort and eight states in the Child Stanford Cohort have non-zero occupancy rates.

VB-HMM identified dynamic functional states, their time evolution and the functional connectivity between nodes of the SN, CEN and DMN in each state (**Fig 4A and 4B**). There were nine states with non-zero occupancy rates and among them three states had rates above 10% (**Fig 4C**). The mean lifetime of the top three high-occupancy states was 10–20 s (**Fig 4D**), confirming temporal persistence over durations much shorter than the length of the scan (480 s). The occupancy rate and mean lifetime of states were not significantly correlated with mean framewise displacement (*q* > 0.05). Additionally, we did not find a state or a subset of states that was consistently related to high or low motion periods across subjects (*q* > 0.05).

#### Children

We repeated the above analyses using data from children. VB-HMM identified seven states with non-zero occupancy rates and among them only three states had rates above 10% (**Fig 4E–4G**). The mean lifetime of the top five high-occupancy states ranged from 10 to 70 s (**Fig 4H**), suggesting persistence of states over greater durations in children compared to adults. Similar to adults, the occupancy rate and mean lifetime of states were not correlated with mean framewise displacement (*q* > 0.05). Additionally, we did not find a state or a subset of states that was consistently related to high or low motion periods across subjects (*q* > 0.05).

### Dynamic functional networks in adults and children

#### Adults

**Fig 5:**
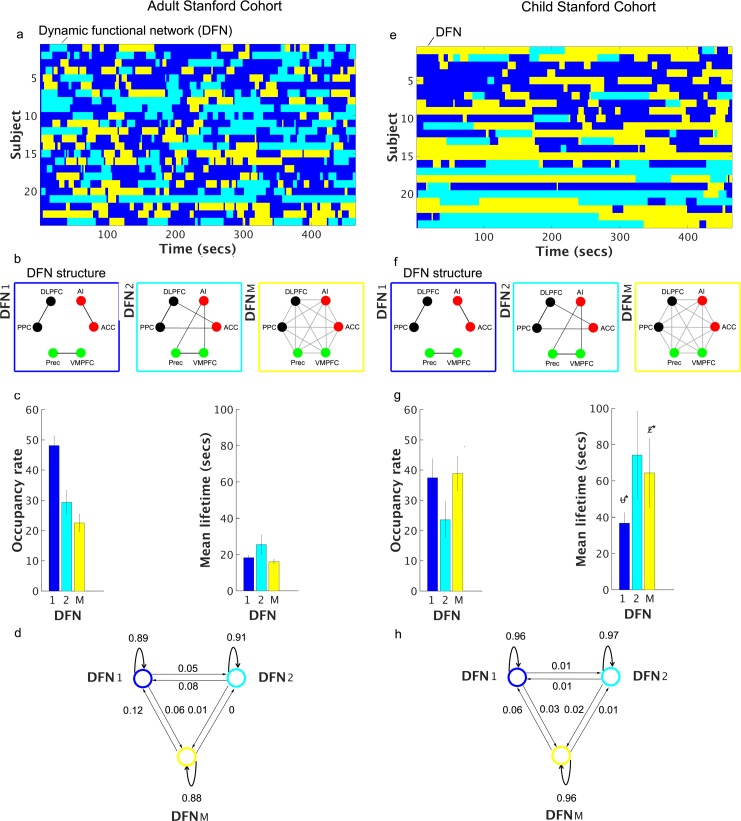
Dynamic functional networks identified in Stanford developmental data. **Adult Stanford Cohort: (a)** Time evolution of merged dynamic networks in each subject; **(b)** Dynamic functional networks (DFNs) for the merged states 1 and 2 and the mixed state; **(c)** Occupancy rates and mean lifetimes of the DFNs; and **(d)** Switching probabilities between the DFNs. **Child Stanford Cohort: (e)** Time evolution of merged dynamic networks in each subject; **(f)** Dynamic functional networks (DFNs) for the merged states 1 and 2 and the mixed state; **(g)** Occupancy rates and mean lifetimes of the DFNs; and **(h)** Switching probabilities between the DFNs. In both cohorts, the most dominant DFN (DFN-1) consists of nodes in the SN, CEN, and DMN, which constitute three independent communities. DFN-1 has an occupancy rate of 48% (SEM: ± 3.2%) with a mean lifetime of 18.3s (SEM: ± 1.2s) for the Adult Stanford Cohort and an occupancy rate of 37% (SEM: ± 6.3%) with a mean lifetime of 18s (SEM: ± 1.2s) for the Child Stanford Cohort. In the second most dominant DFN (DFN-2), which is identified in both datasets, the nodes of the CEN and DMN are connected to the nodes of the SN. DFN-2 had an occupancy rate of 29% (SEM: ± 3.9%) with a mean lifetime of 25.5s (SEM: ± 5.3s) in the Adult Stanford Cohort and an occupancy rate of 24% (SEM: ± 6.0%) with a mean lifetime of 74s (SEM: ± 24.0s) in the Child Stanford Cohort. In both cohorts, the self-transition probabilities from a DFN to itself are high (> 0.88), while the transitions between DFNs are low (< 0.1).

We identified two DFNs with distinct community structures (**Fig 5A and 5B**). In DFN-1, the SN, CEN and DMN were disconnected from each other and formed three independent communities (States 2, 3, 7, and 8 in **S8 Fig**). This disconnected network configuration had a combined occupancy rate of 48% ± 3.2% and a mean lifetime of 18.3s ± 1.2s (**Fig 5C**). The network configuration that showed the next highest occupancy rate was one in which the CEN and DMN were connected with the SN (States 1 and 6 in **S8 Fig**). This connected network configuration (DFN-2 in **Fig 5B**) had an occupancy rate of 29% ± 3.9% and a mean lifetime of 25.5s ± 5.3s (**Fig 5C**). All other network configurations had occurrence rates of 11% or less (**S8 Fig**).

#### Children

We repeated the same analysis using data from children. We identified two DFNs with district community structures (**Fig 5E and 5F**). In DFN-1, the SN, CEN and DMN were disconnected from each other and formed three independent communities (States 3 and 4 in **S9 Fig**). This disconnected network configuration had a combined occupancy rate of 37% ± 6.3% and a mean lifetime of 18s ± 1.2s (**Fig 5G**). The network configuration that showed the next highest occupancy rate was one in which the CEN and DMN were connected with the SN (State 1 in **S9 Fig**). This connected network configuration (DFN-2 in **Fig 5F**) had a combined occupancy rate of 24% ± 6.0% with a mean lifetime of 74 s ± 24 s (**Fig 5G**). All other network configurations had occurrence rates of 11% or less (**S9 Fig**).

The above analysis demonstrates that adults and children have two common dominant DFNs with identical network structures. We used this commonality to probe the maturation of dynamic brain networks in terms of the differences in occupancy rates, mean lifetimes, and switching probabilities between these common DFNs.

### Developmental changes in occupancy rates and mean lifetimes of dynamic functional networks

**Fig 6:**
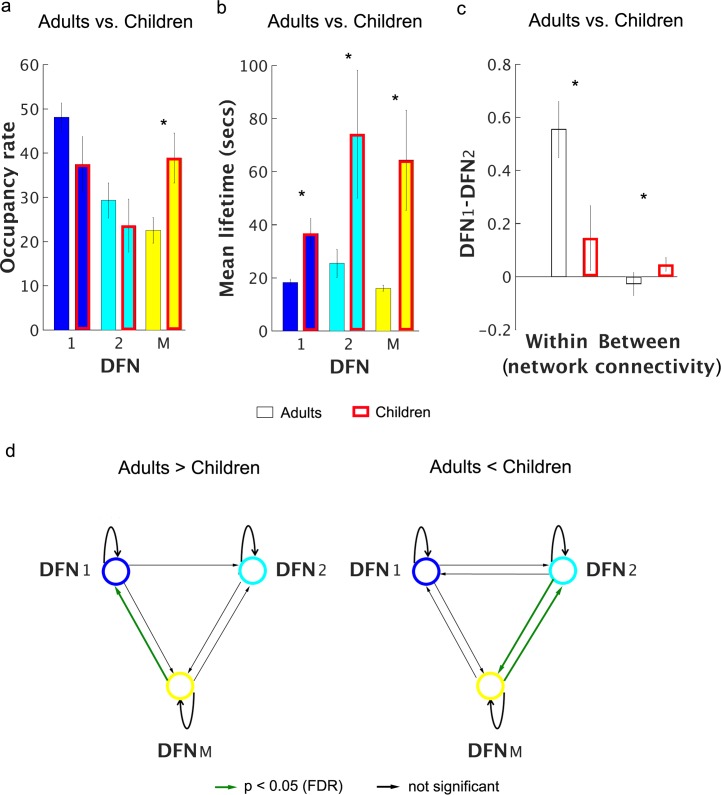
Developmental changes in occupancy rate, mean lifetime, state transition probabilities, and connectivity of DFNs in Stanford developmental data. **(a)** Occupancy rates of DFNs in adults and children did not differ (p > 0.05, FDR corrected). **(b)** Mean lifetimes of DFNs are significantly higher in children compared to adults (p < 0.05, FDR corrected). The higher mean lifetimes in children suggest that DFNs persist longer in children compared to adults. **(c)** DFN connectivity is weaker and less differentiated in children relative to adults. Adults showed strong differences in the connectivity of within- and cross-network links spanning the three static networks (SN, CEN, and DMN), whereas children did not (*p* < 0.002). **(d)** The probability of within-state (DFN) transitions was not significantly different between the two groups (p > 0.05, FDR corrected). State transition probabilities from DFN-2 and DFN-m to DFN-1 were significantly higher in adults compared to children (p < 0.05, FDR corrected). State transition probabilities from DFN-2 to DFN-m and from DFN-m to DFN-2 were greater in children compared to adults (*p* < 0.05, FDR corrected). These findings demonstrate that compared to children, adults switch back more frequently to DFN-1, in which the SN, DMN, and CEN are completely segregated from each other.

To investigate whether DFN occupancy rates and mean lifetimes differ between children and adults, we focused on DFN-1 and DFN-2, the two dominant DFNs with identical community structures in adults and children that together account for about 77% occupancy rates in both groups. Network configurations corresponding to all other functional states were combined into DFN-M. The mean lifetimes, but not the occupancy rates, of all three DFNs were significantly greater in children compared to adults (*p* < 0.05, FDR corrected) (**Fig 6A and 6B**). These findings indicate that children tend to persist longer in the same DFN than adults, as illustrated by the time evolution of the three DFNs (**Fig 5A and 5F**). Below we further investigate this pattern of developmental differences in terms of transition probabilities between DFNs.

### Developmental changes in transition probability between dynamic functional networks

To further investigate whether children tend to stay in one DFN configuration longer than adults, we computed transition probabilities in children and adults and compared them between the groups. The probability of within-DFN transitions was not significantly different between the two groups (*p* > 0.05, FDR corrected). However, transition probabilities to the fully disconnected SN-CEN-DMN configuration (DFN-1) from both connected network configurations (DFN-2 and DFN-M) were significantly higher in adults compared to children (*p* < 0.05, FDR corrected) (**Fig 6D**). In contrast, children showed a higher probability of switching between the two connected network configurations *(p* < 0.05, FDR corrected). These findings demonstrate that, compared to children, adults switch back more frequently to DFN-1, in which the SN, DMN and CEN are completely segregated from each other.

### Developmental changes in dynamic functional network connectivity

Finally, to investigate how dynamic functional connectivity matures with age we compared the strength of DFN connectivity assessed using within- and cross-network links as described above. In this analysis, we further excluded participants with DFN connectivity beyond 3 standard deviations from their specific group or for whom both DFNs were not present. After exclusion, our sample consisted of 22 adults and 16 children. We found a significant three-way interaction between DFN (DFN-1 vs. DFN-2), link type (within- vs. cross-network), and participant groups (children vs. adults) (*F*_*1*,*36*_ = 10.99, *p* = 0.002) (**Fig 6C**), such that DFN-1 and DFN-2 configurations differed in connection strength by link type in adults (*F*_*1*,*21*_ *=* 119.5, *p* < 0.001) but not in children (*F*_*1*,*15*_ = 0.491, *p* = 0.494). These results demonstrate that DFN connectivity is weaker and less differentiated in children relative to adults.

## Discussion

The main scientific aims of our study were to (1) investigate the temporal properties of dynamic functional connectivity between the SN, CEN and DMN, three core neurocognitive networks implicated in a wide range of goal directed behaviors [12,15,16,26,48,49], and (2) investigate how the temporal properties of dynamic functional connectivity between these core networks change from childhood to adulthood. To accomplish this, we first developed a novel Bayesian HMM (VB-HMM) model for quantifying dynamic changes in functional connectivity. A variational Bayes approach for estimating latent states and unknown HMM model parameters allowed us to overcome weaknesses associated with conventional methods and to investigate dynamic changes in intrinsic functional connectivity between three networks, which have previously only been investigated using static network analysis. VB-HMM allowed us to quantify the temporal evolution of distinct brain states and probe the dynamic functional organization of the SN, CEN and DMN in an analytically rigorous manner. Contrary to previous observations based on static time-averaged connectivity analysis [20,50], we found that temporal coupling between the SN, CEN and DMN varies considerably over time and that these networks exist in a completely segregated state only intermittently with relatively short mean lifetimes. VB-HMM also revealed immature and inflexible dynamic interactions between the SN, CEN and DMN characterized by higher mean lifetimes in individual states and reduced transition probability between states, in children relative to adults.

VB-HMM is a novel machine learning approach for identifying dynamic changes in functional brain connectivity. VB-HMM has several advantages over existing methods [6,9,29,51,52]: (i) the automated estimation of latent states and their temporal evolution; (ii) estimation of posterior probabilities of latent states and model parameters; (iii) selection of models based on a trade-off between the model complexity and fit of the data, thereby reducing overfitting; (iv) use of sparsity constraints resulting in pruning of weak states without having to specify the number of states *a priori*; and (v) a generative model that has the potential to provide a more mechanistic understanding of human brain dynamics. Our approach also overcomes weaknesses of existing HMM methods that are based on a maximum likelihood estimation approach and require *a priori* specification of the number of hidden states. Furthermore, in contrast to conventional HMM methods, VB-HMM can discover dynamic changes in states based on signal mean or covariance or both. This flexibility can be useful in uncovering latent brain dynamics during cognitive task processing, where states typically differ in both signal mean and covariance, as well as rs-fMRI, where states are better characterized by changes in covariance rather than mean signal levels. In applications to rs-fMRI, as in the present study, this is accomplished in VB-HMM by setting the prior hyperparameter value *λ*_*k*_ = 1000 for each state k. This choice forces the posterior mean values for each state (*μ*_*k*_) close to prior mean (which is zero) (Equation S.10) and ensure that states are characterized by differences in the covariance matrices (Σ_*k*_), but not the mean (*μ*_*k*_). Another advantage of our Bayesian approach is that the covariance (or inverse covariance) estimates are regularized and the extent of regularization is determined by the data (Eqs S11–S.13). This regularization ensures that the covariance matrices are full rank and therefore invertible to estimate partial correlations. Such regularized estimation is not possible with maximum likelihood approaches. Our simulations using three different simulation models demonstrate that VB-HMM can accurately discover the number of states, their temporal evolution, the transition probabilities between states and dynamic connectivity patterns associated with each state (see **S1 Text** for details).

We next used VB-HMM to characterize the temporal evolution of dynamic brain states in two independent cohorts of adult participants from the HCP. VB-HMM identified multiple stable states in both cohorts of participants. The observation that the number of states is strictly greater than one is consistent with previous results demonstrating that the rs-fMRI time series is not stationary[29,53]. Importantly, VB-HMM identified similar patterns of stable brain states in both cohorts and provided reliable and replicable estimates of occupancy rates, mean lifetimes, and state transition probabilities associated with each brain state. Although VB-HMM identified 16–19 states in both adult cohorts, only three states had occupancy rates greater than 10% (**Fig 2C and 2G**), and these states demonstrated the highest mean lifetimes. However, even these dominant states had short mean lifetimes ranging from 7–10 s, demonstrating that brain states are temporally persistent over durations far shorter than the length of a typical rs-fMRI scan session. These features were observed in both adult cohorts, demonstrating the robustness of our findings. Furthermore, analysis of the state transition probability indicated that each state had the highest probability of transitioning to itself rather than other states (**Fig 2D and 2H)**, suggesting that temporal stability of individual states does occur. Taken together, these results demonstrate the existence of dynamic, yet stable, brain states in rs-fMRI and identify distinct connectivity patterns associated with each state. We suggest that this balance of temporal stability and dynamic connectivity is a fundamental principle of brain organization.

By construction, VB-HMM states are characterized by distinct patterns of inter-node connectivity (**Figs 2 and 3**). To test specific hypotheses related to the dynamic interactions between the SN, CEN and DMN and interpret the neurobiological relevance of connectivity profiles, we identified dynamic functional connectivity profiles associated with the three previously known static networks. To accomplish this we applied modularity-based community detection algorithms [36] on the functional connectivity matrix estimated by VB-HMM for each state (**Fig 1B**). This analysis revealed that, in some cases, states with non-identical connectivity matrices had similar overall community structures (**S5, S6, S8 and S9 Figs).** For example, multiple states (**S5 and S6 Figs)** demonstrated a pattern in which the SN, CEN and DMN formed separate, segregated communities, reminiscent of the static functional networks previously identified by independent components analysis [50]. We next combined states with identical community structures into dynamic functional networks (DFNs) and examined the temporal properties of segregated and non-segregated DFNs as well as the dynamic interactions between key nodes of the SN, CEN and DMN.

The SN, CEN and DMN formed separate communities and were segregated from each other (DFN-1 in **Fig 3**) approximately 31% of the time (31% and 27% in Cohorts 1 and 2, respectively). In this case, all three networks maintained their within-network connectivity structure–AI and ACC nodes of the SN were connected with each other, PMC and VMPFC nodes of the DMN were connected with each other, and DLPFC and PPC nodes of the CEN were connected with each other. Crucially, VB-HMM also revealed that this DFN had a mean lifetime of about 7–10 s (8.3 s and 8.8 s in Cohorts 1 and 2, respectively) (**Fig 3C and 3G**). These findings suggest that although this particular DFN configuration is a prominent feature of SN, CEN and DMN organization, it has a relatively short lifetime.

The second dominant DFN identified by VB-HMM had a community structure in which the CEN and DMN were interconnected in one community, while the SN nodes remained segregated from the CEN and DMN, forming an independent network (DFN-2 in **Fig 3**). This DFN configuration had occurrence rates of 36% and 18% in Cohorts 1 and 2, respectively (**Fig 3**). The remaining states had distinct DFN configurations (**S5 and S6 Figs**), with varying levels of cross-network interactions, but their occurrence rates were lower and not consistent across the two cohorts. Previous work from our lab [12] [39] and recent work by other labs [54,55] has indicated that the SN plays a critical role in switching between the DMN and the CEN. Our results suggest that this switching is transient (i.e. doesn’t persist for a long time) and may occur not very frequently.

Finally, analysis of the switching probability between DFNs revealed that each DFN had a high probability (0.91 in Cohort 1 and 0.93 in Cohort 2) of making self-transitions (**Fig 3D and 3H)**. Thus, as with individual brain states, the two dominant DFN configurations (DFN-1 and DFN-2 in **Fig 3**) were stable over time but persistent only for short time intervals. Taken together, these findings identify key features of dynamic functional interactions associated with the SN, CEN and DMN and confirm that the static segregated networks previously identified using independent component analysis occur only about 30% of the time.

The organization of brain networks in adults is shaped by years of development, learning and brain plasticity [5]. Previous studies using static connectivity analysis have pointed to changing topological organization of connections with age [56–61]. More specifically, it has been suggested that interactions between the SN, CEN and DMN are immature in children [26,28], but their dynamical temporal properties are not known because previous analyses have assumed that brain networks are static over time. To address this gap in knowledge, we used VB-HMM to investigate how dynamic functional interactions between the SN, CEN and DMN mature from childhood to adulthood. VB-HMM revealed significant differences in key temporal properties, such as mean lifetime and state transition probabilities, between children and adults and provides a new level of detail regarding immature brain dynamics in childhood.

To test the hypothesis that dynamic functional interactions between the SN, CEN and DMN are different in children, we first identified two common dominant DFNs with identical network structure in both children and adults. We used this commonality to probe the maturation of dynamic brain networks using measures of occupancy rate, mean lifetime and switching probabilities derived using VB-HMM. VB-HMM identified two DFN configurations with the same community structure in both groups: DFN-1, in which the SN, CEN and DMN were segregated from each other and DFN-2, in which the AI and ACC nodes of the SN were decoupled from each other and showed significant cross-network interactions with the DMN and CEN, respectively (**Fig 5B and 5F**). Critically, the network structures of the DFN-2 were different between the HCP Adult cohorts (**Fig 3B and 3F**) and Stanford Adult cohort (**Fig 5B**). The differences may have arisen from the slower sampling rate in the Stanford data which used a standard TR = 2 seconds compared to the faster TR = 0.73 seconds used in the HCP data. Critically, patterns were consistent within scanners–the first and second DFNs were identical in the two HCP cohorts and in the two Stanford cohorts. Analysis of connectivity profiles across nodes of the SN, CEN and DMN showed that the two DFNs were less differentiated in children relative to adults (**Fig 6C**). Critically, the mean lifetimes of the two common DFN configurations (DFN-1 and DFN-2) were significantly greater in children compared to adults (**Fig 5G**) suggesting that immature brain network organization is characterized by greater dwelling time in specific network configurations.

Analysis of transition probabilities further revealed that the likelihood of transitions into the configuration in which the SN, CEN and DMN were completely segregated from each other was significantly lower in children compared to adults (**Fig 6D**). In contrast, children showed a higher likelihood of switching between non-segregated network configurations (**Fig 6D**). These findings support the notion that relative to those of adults, children’s brains are less flexible and less likely to switch to the segregated DFN configuration from other network configurations. Taken together, these findings demonstrate that children have less flexible dynamic cross-network interactions, characterized by reduced switching between distinct brain states and longer persistence in specific network configurations.

In summary, we developed a novel Bayesian HMM (VB-HMM) approach for estimating the temporal properties of dynamic functional networks in fMRI data and applied it to characterize time-varying connectivity of the SN, DMN, and CEN, three neurocognitive networks that play a crucial role in human cognition. VB-HMM uncovered latent states, dynamic functional connectivity and state transition probabilities associated with these three networks, thereby revealing transient dynamic functional networks (DFNs) that allow for flexible within and cross-network interactions. In adults, VB-HMM revealed that the SN, CEN and DMN–systems that were previously characterized only by static network analysis–were in a segregated, disconnected state, only about 30% of the time with mean lifetimes of 7–10 s. VB-HMM also revealed that dynamic functional interactions between the SN, CEN and DMN are weaker and immature in children. Critically, the uncovered brain dynamics were not related to individual differences in age and in-scanner micro-movements. Our computational techniques provide new insights into the dynamic functional organization of the SN, DMN and CEN and their maturation with development. More generally, our computational approach may be useful for investigating the dynamic aspects of functional brain organization in neurodevelopmental and psychiatric disorders, including autism, schizophrenia and mood disorders[27].

## Supporting Information

S1 Fig Regions of Interest (ROIs). ROIs are identified using Independent Component Analysis (ICA) applied on an independent dataset from a previously-published study. ROIs include right anterior insula (AI) and anterior cingulate cortex (ACC) in the Salience Network (SN); right dorsolateral prefrontal cortex (DLPFC) and posterior parietal cortex (PPC) in the Central Executive Network (CEN); and Precuneus (Prec) and ventral medial prefrontal cortex (VMPFC) in the Default Mode Network (DMN).
(TIFF)

S2 Fig Validation of VB-HMM–Simulation Model 1. (**a**) State transition used to generate the simulated dataset. (**b**) Probability of each state at each time point computed by applying VB-HMM to the simulated dataset. (**c**) State transition uncovered by VB-HMM.
(TIFF)

S3 Fig Validation of VB-HMM–Simulation Model 2. (**a**) State transition used to generate the simulated dataset. (**b**) Probability of each state at each time point computed by applying VB-HMM to the simulated dataset. (**c**) State transition uncovered by VB-HMM.
(TIFF)

S4 Fig Validation of VB-HMM–Simulation Model 3. (**a**) State transition used to generate the simulated dataset. (**b**) Probability of each state at each time point computed by applying VB-HMM to the simulated dataset. (**c**) State transition uncovered by VB-HMM.
(TIFF)

S5 Fig Dynamic functional networks for each state discovered by VB-HMM in Adult HCP Cohort 1: Dynamic Functional networks are obtained by applying a community detection algorithm on partial correlations estimated (Fig 3B) by VB-HMM. States are ordered from the highest to lowest occupancy rates. Among 25 states only 16 have nonzero occupancy rates.
(TIFF)

S6 Fig Dynamic functional networks for each state discovered by VB-HMM in Adult HCP Cohort 2: Dynamic Functional networks are obtained by applying a community detection algorithm on partial correlations estimated (Fig 3F) by VB-HMM. States are ordered from the highest to lowest occupancy rates. Among 25 states only 18 have nonzero occupancy rates.
(TIFF)

S7 Fig **Dynamic functional network connectivity in Adult HCP Cohort 1 and Adult HCP Cohort 2:** (**a**) In Cohort 1, there was a found a significant interaction DFN and link type (*F*_*1*,*19*_ = 4.943, *p* = 0.039) such that connectivity of cross-network links was greater in DFN-2 compared to DFN-1 (*p* < 0.001) while no significant difference was observed between DFN-1 and DFN-2 for within-network links (*p* = 0.461). (**b**) In Cohort 2, similar to Cohort 1, there was a found a significant interaction DFN and link type (*F*_*1*,*19*_ = 40.87, *p* < 0.001), such that the strength of cross-network links was greater in DFN-2 compared to DFN-1 (*p* < 0.001) while the reverse was true for within-network links (*p* < 0.001). These results demonstrate that the two DFNs differ significantly in their connectivity profiles.
(TIF)

S8 Fig Dynamic functional networks for each state discovered by VB-HMM in Adult Stanford Cohort: Dynamic Functional networks are obtained by applying a community detection algorithm on partial correlations estimated (Fig 4B) by VB-HMM. States are ordered from the highest to lowest occupancy rates. Among 25 states 9 only states have nonzero occupancy rates.
(TIFF)

S9 Fig Dynamic functional networks for each state discovered by VB-HMM in Child Stanford Cohort: Dynamic Functional networks are obtained by applying a community detection algorithm on partial correlations estimated (Fig 4F) by VB-HMM. States are ordered from the highest to lowest occupancy rates. Among 25 states only 8 states have nonzero occupancy rates.
(TIFF)

S1 Table Participant demographics and motion parameters during fMRI scanning (DOCX)

S1 File Supplementary Materials and Methods (DOCX)
