# Supplementary figures and images for: Temporal Dynamics and Developmental Maturation of Salience, Default and Central-Executive Network Interactions Revealed by Variational Bayes Hidden Markov Modeling
Source: PLoS Comput Biol. 2016 Dec 13;12(12):e1005138. doi: 10.1371/journal.pcbi.1005138 (PMC5154470; doi:10.1371/journal.pcbi.1005138)

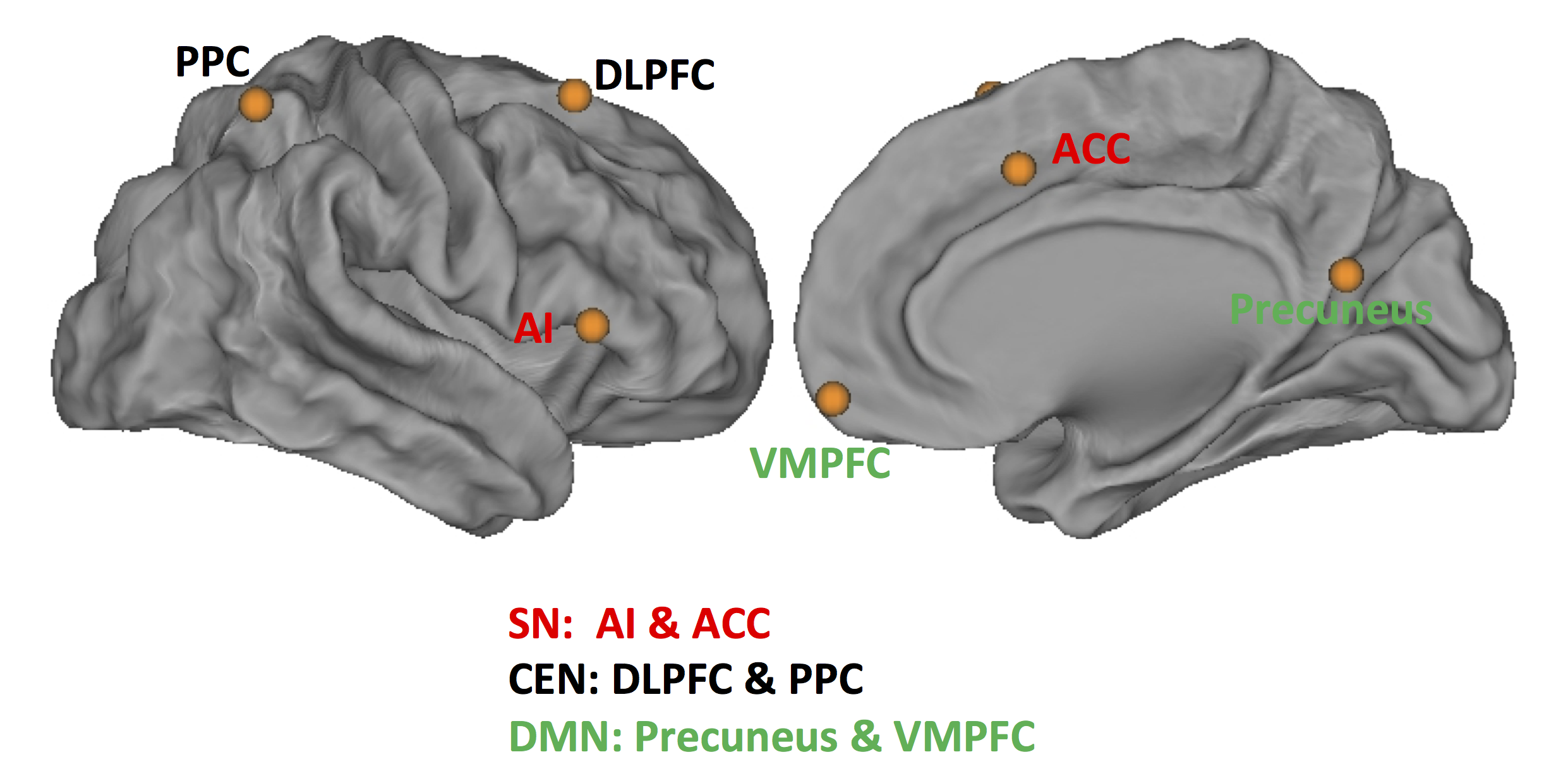

Supplement: S1 Fig — ROIs are identified using Independent Component Analysis (ICA) applied on an independent dataset from a previously-published study. ROIs include right anterior insula (AI) and anterior cingulate cortex (ACC) in the Salience Network (SN); right dorsolateral prefrontal cortex (DLPFC) and posterior parietal cortex (PPC) in the Central Executive Network (CEN); and Precuneus (Prec) and ventral medial prefrontal cortex (VMPFC) in the Default Mode Network (DMN). (TIFF) [file pcbi.1005138.s001.tiff]

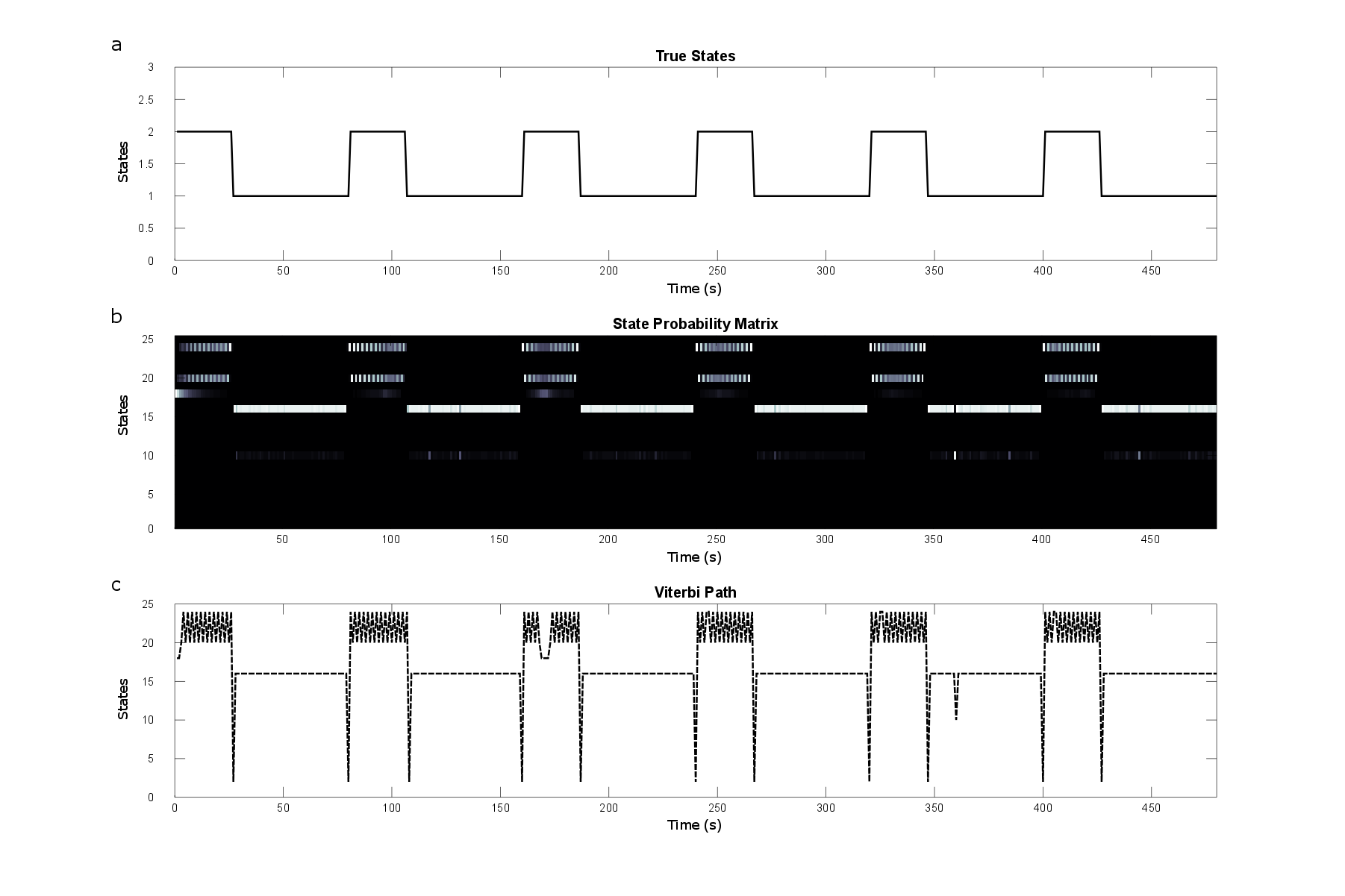

Supplement: S2 Fig — (a) State transition used to generate the simulated dataset. (b) Probability of each state at each time point computed by applying VB-HMM to the simulated dataset. (c) State transition uncovered by VB-HMM. (TIFF) [file pcbi.1005138.s002.tiff]

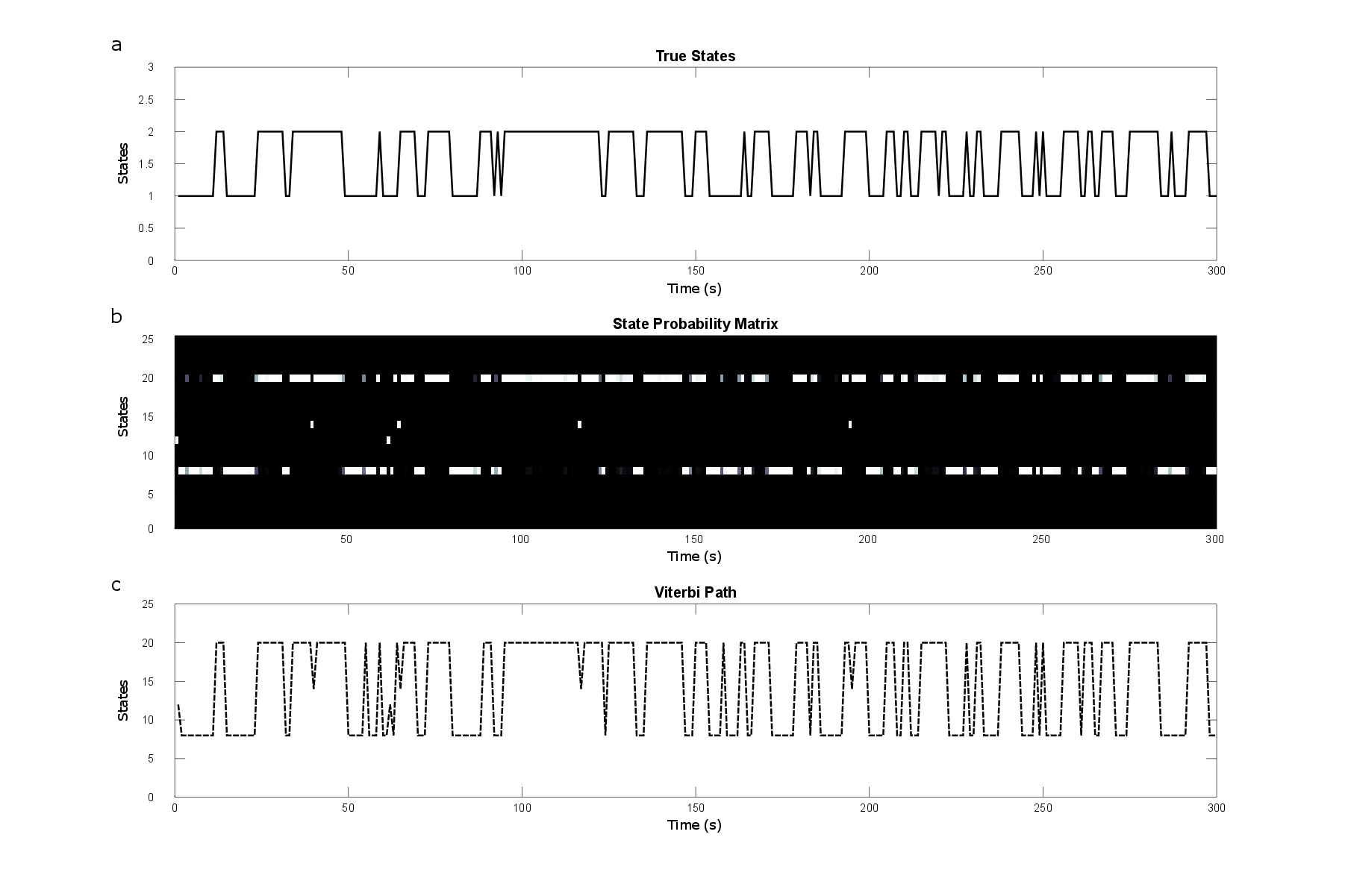

Supplement: S3 Fig — (a) State transition used to generate the simulated dataset. (b) Probability of each state at each time point computed by applying VB-HMM to the simulated dataset. (c) State transition uncovered by VB-HMM. (TIFF) [file pcbi.1005138.s003.tiff]

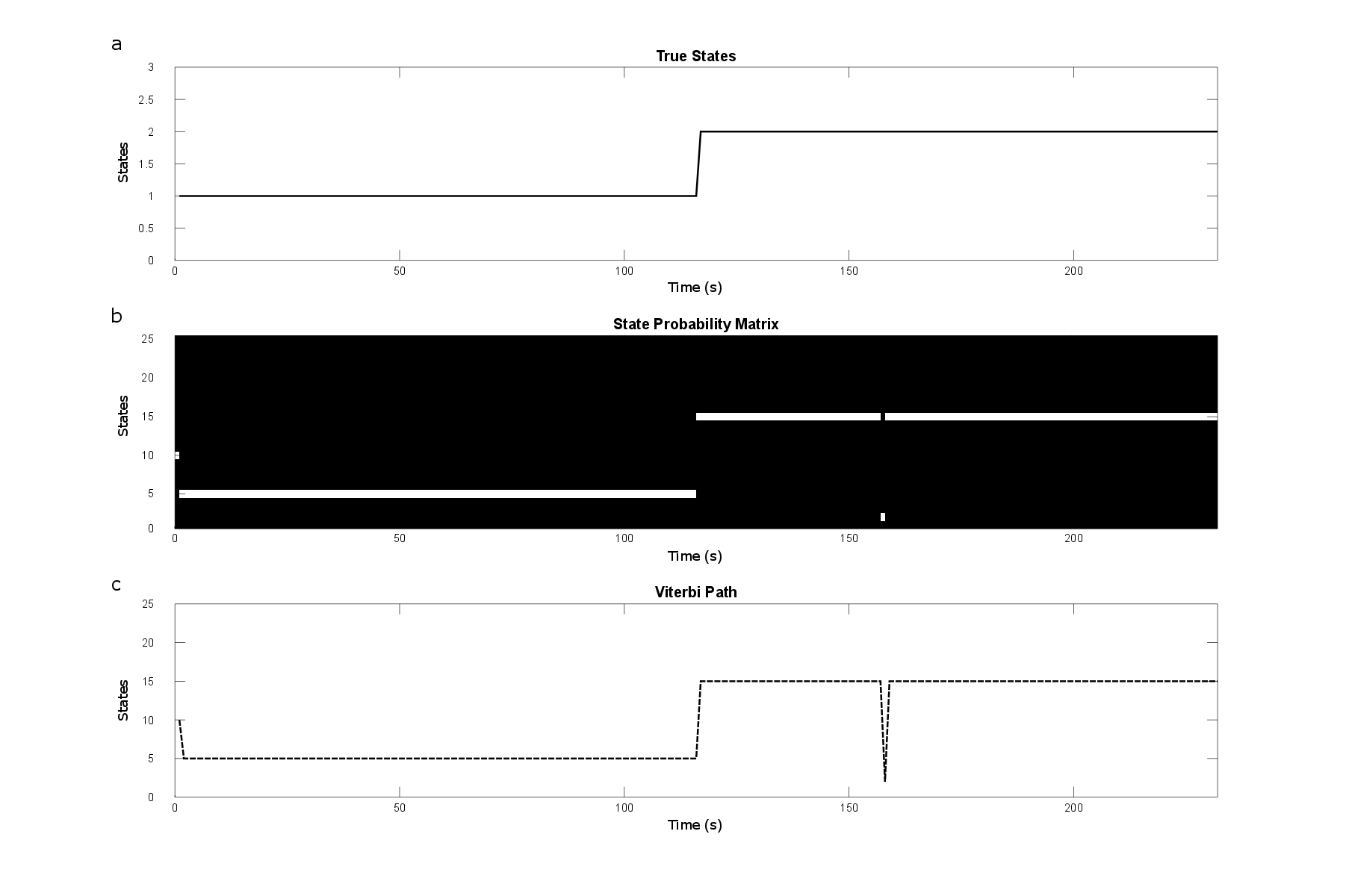

Supplement: S4 Fig — (a) State transition used to generate the simulated dataset. (b) Probability of each state at each time point computed by applying VB-HMM to the simulated dataset. (c) State transition uncovered by VB-HMM. (TIFF) [file pcbi.1005138.s004.tiff]

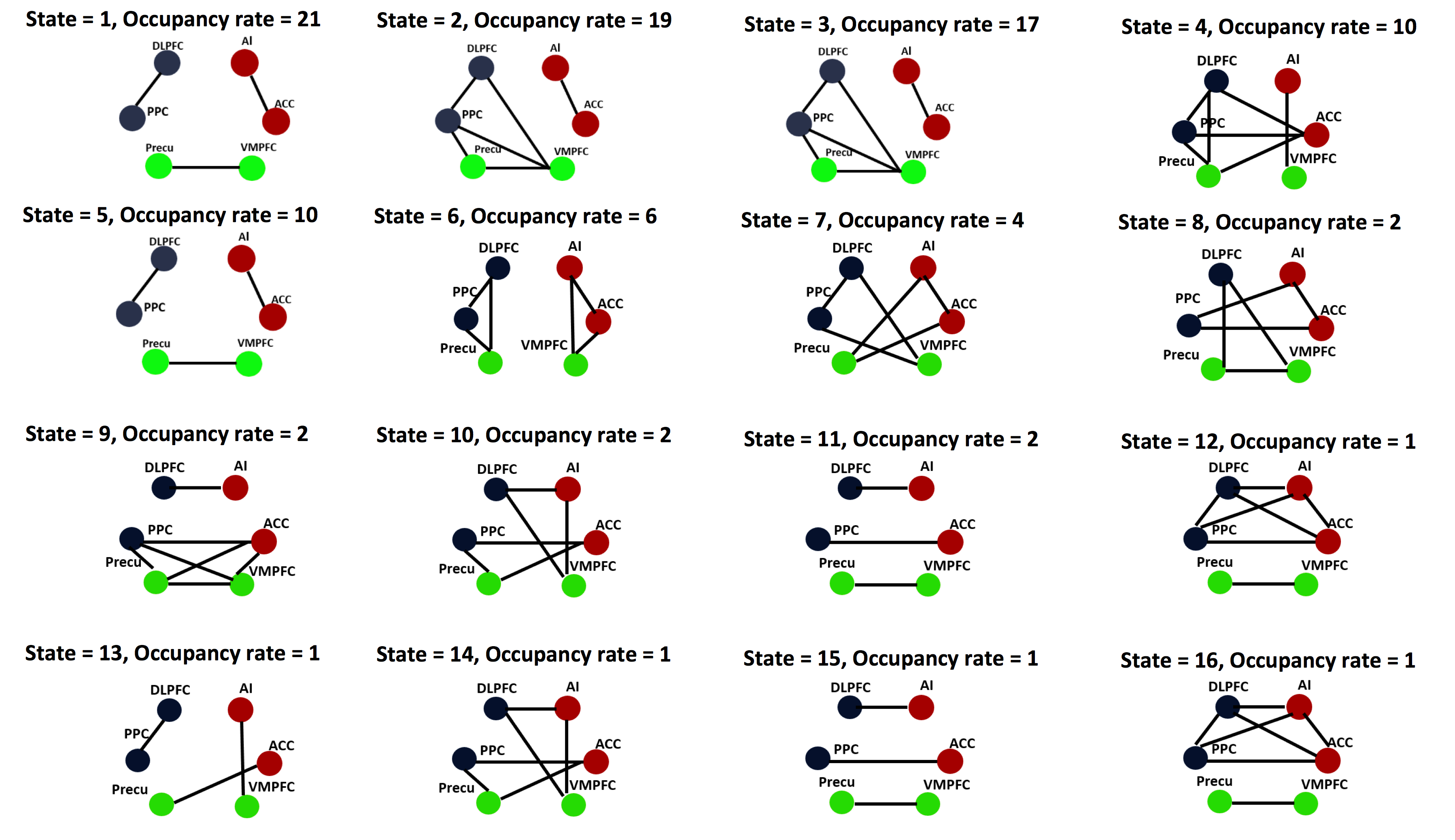

Supplement: S5 Fig — States are ordered from the highest to lowest occupancy rates. Among 25 states only 16 have nonzero occupancy rates. (TIFF) [file pcbi.1005138.s005.tiff]

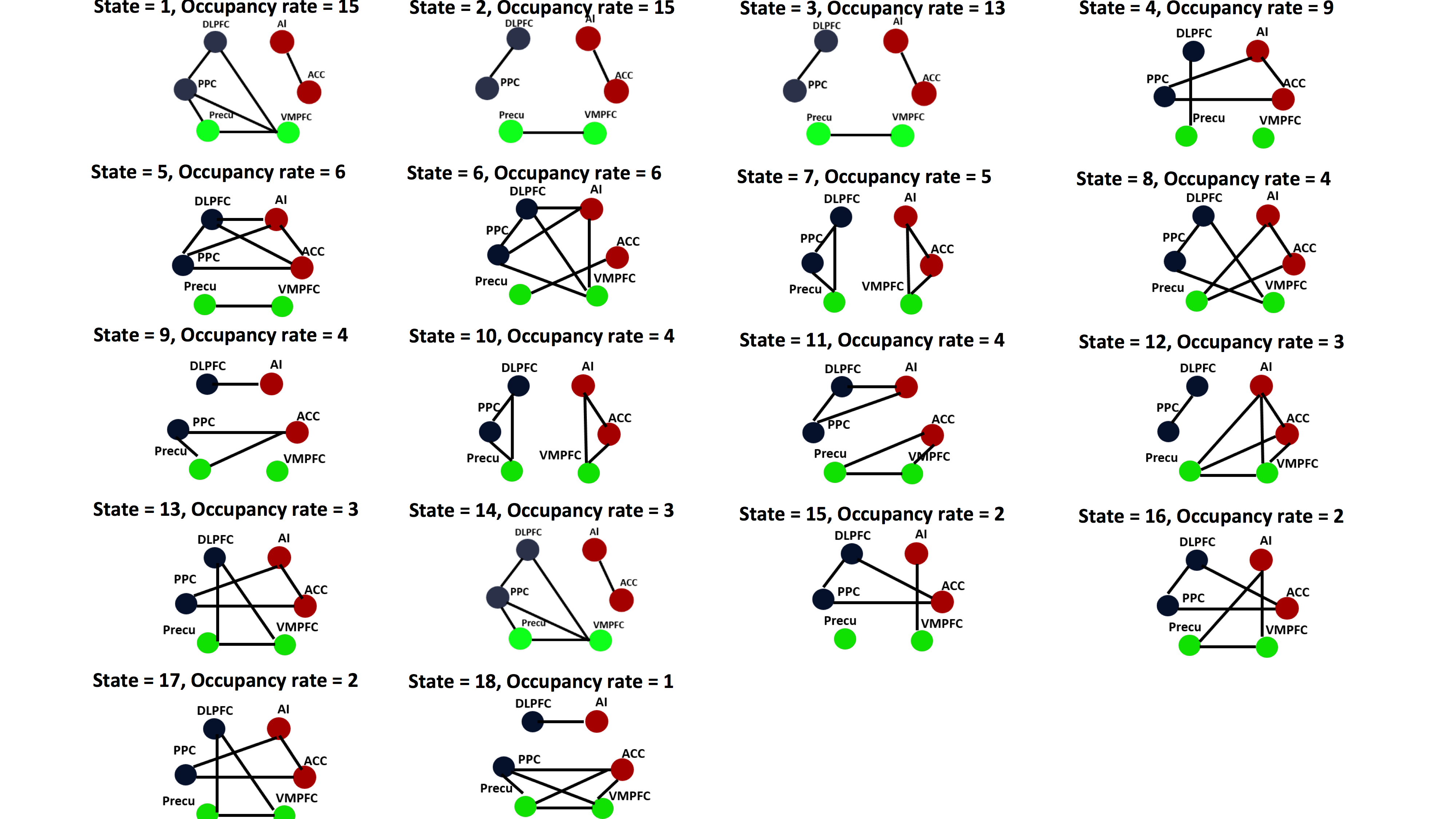

Supplement: S6 Fig — States are ordered from the highest to lowest occupancy rates. Among 25 states only 18 have nonzero occupancy rates. (TIFF) [file pcbi.1005138.s006.tiff]

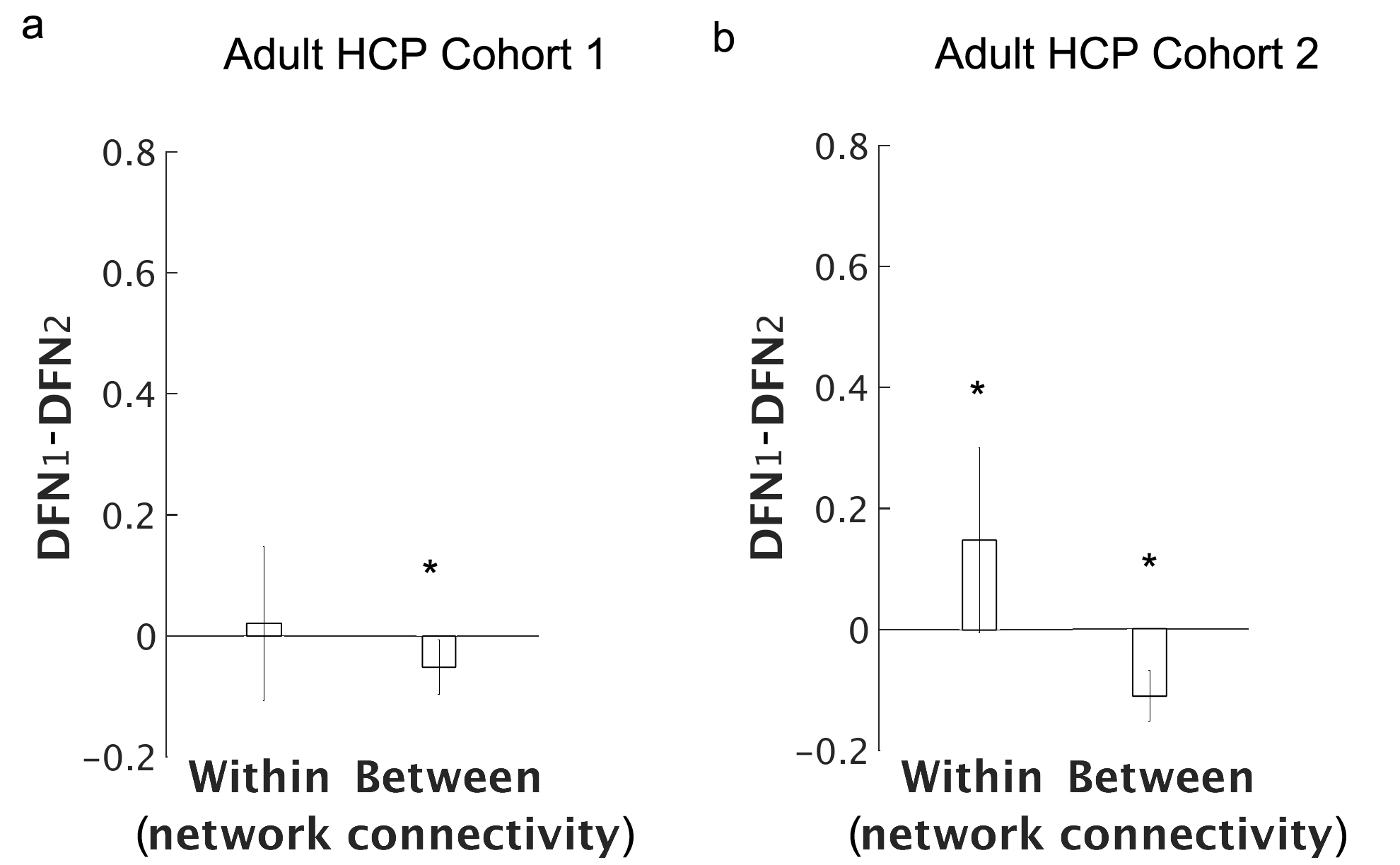

Supplement: S7 Fig — Dynamic functional network connectivity in Adult HCP Cohort 1 and Adult HCP Cohort 2: (a) In Cohort 1, there was a found a significant interaction DFN and link type (F1,19 = 4.943, p = 0.039) such that connectivity of cross-network links was greater in DFN-2 compared to DFN-1 (p < 0.001) while no significant difference was observed between DFN-1 and DFN-2 for within-network links (p = 0.461). (b) In Cohort 2, similar to Cohort 1, there was a found a significant interaction DFN and link type (F1,19 = 40.87, p < 0.001), such that the strength of cross-network links was greater in DFN-2 compared to DFN-1 (p < 0.001) while the reverse was true for within-network links (p < 0.001). These results demonstrate that the two DFNs differ significantly in their connectivity profiles. (TIF) [file pcbi.1005138.s007.tif]

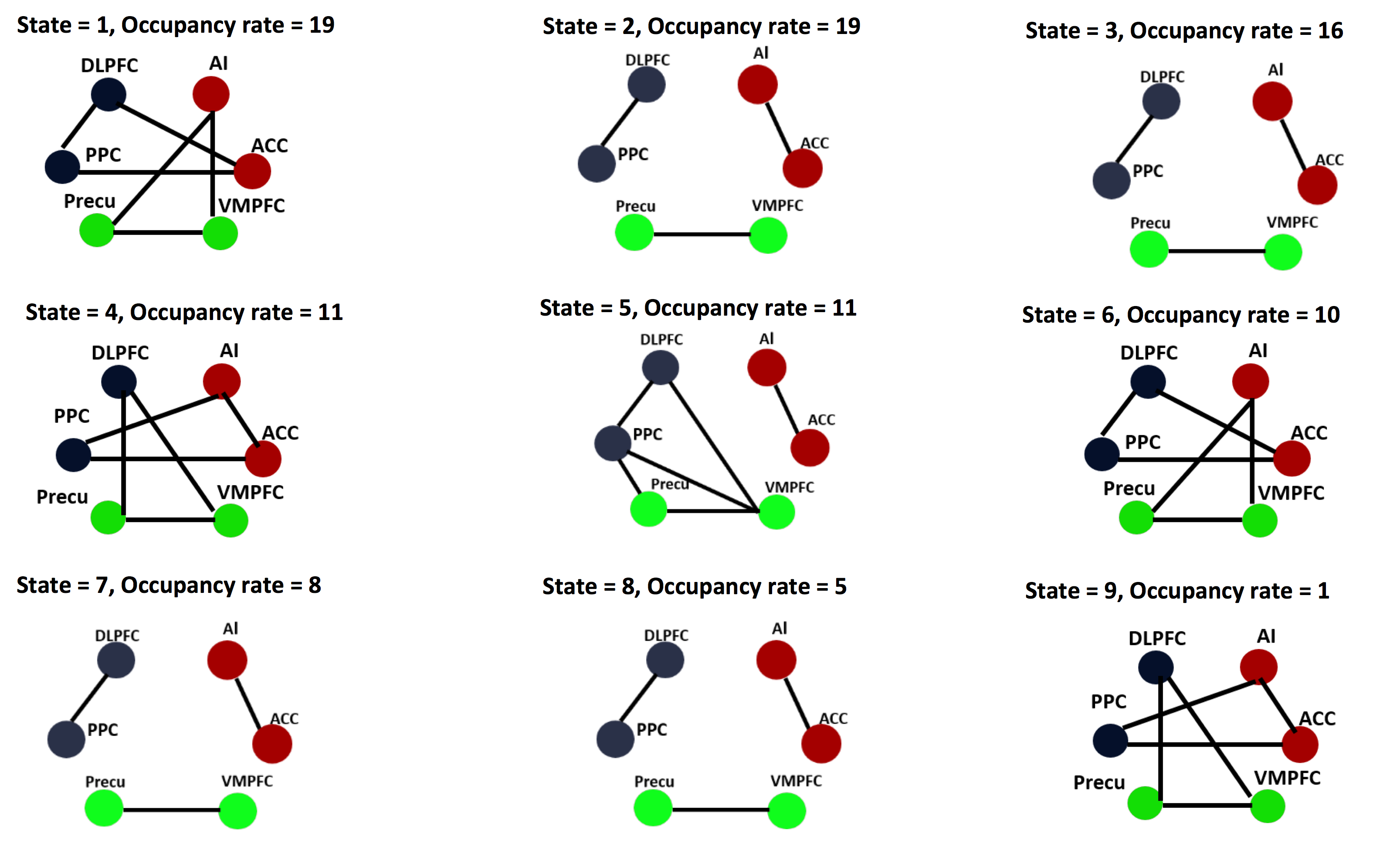

Supplement: S8 Fig — States are ordered from the highest to lowest occupancy rates. Among 25 states 9 only states have nonzero occupancy rates. (TIFF) [file pcbi.1005138.s008.tiff]

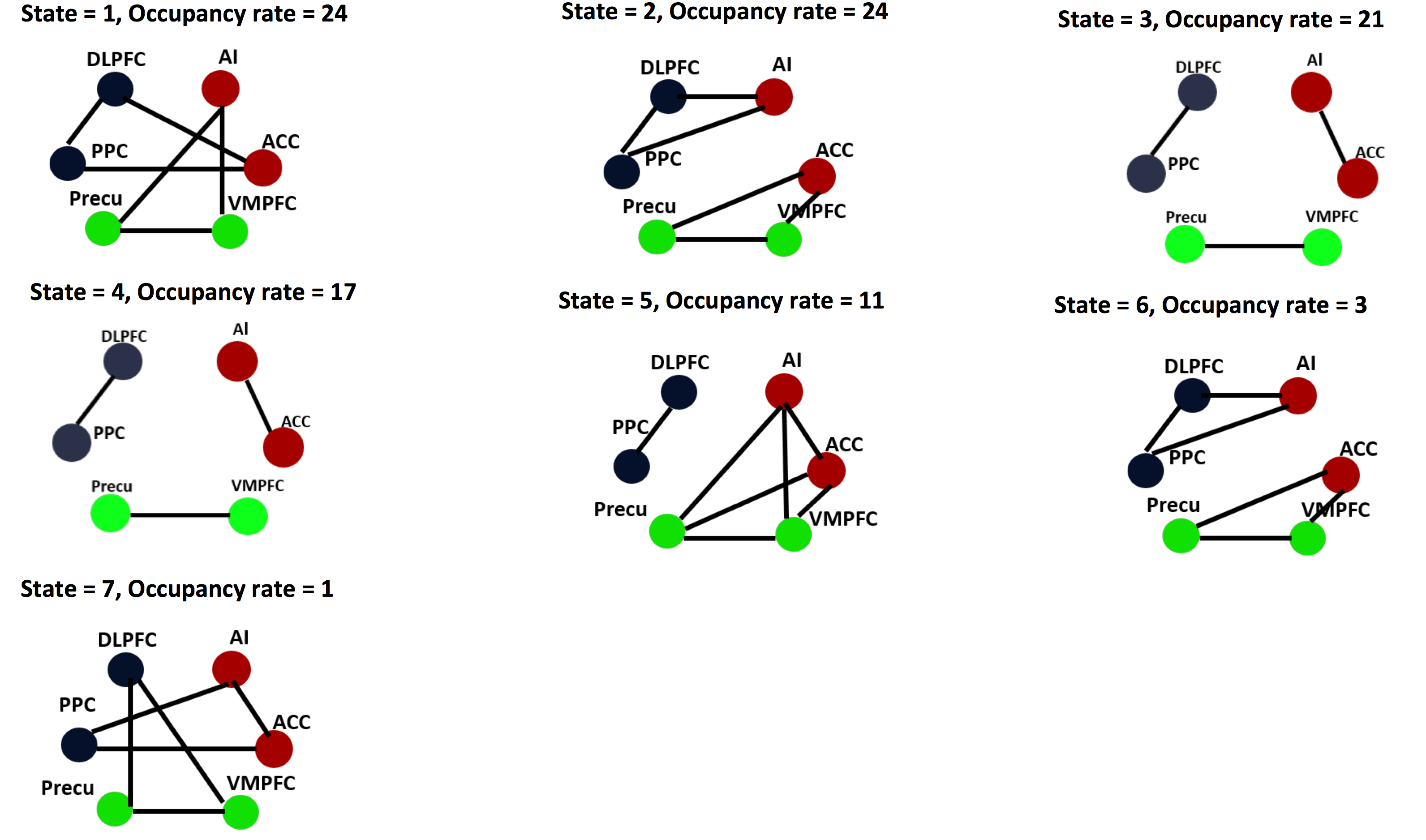

Supplement: S9 Fig — States are ordered from the highest to lowest occupancy rates. Among 25 states only 8 states have nonzero occupancy rates. (TIFF) [file pcbi.1005138.s009.tiff]
