# Supplementary material for: Temporal Dynamics and Developmental Maturation of Salience, Default and Central-Executive Network Interactions Revealed by Variational Bayes Hidden Markov Modeling
Source: PLoS Comput Biol. 2016 Dec 13;12(12):e1005138. doi: 10.1371/journal.pcbi.1005138 (PMC5154470; doi:10.1371/journal.pcbi.1005138)
Supplement: S1 Table — (DOCX) [file pcbi.1005138.s010.docx]

**Table S1. Participant demographics and motion parameters during fMRI scanning**

|  | | Adults (*n* =24) | Children (*n* = 24) | Sig. |  |
| --- | --- | --- | --- | --- | --- |
| Mean age | | 20.56 (range: 19 - 22) | 8.11 (range: 7 - 9) | *p* < 0.001 |  |
| Gender | | 12 M / 12 F | 12 M / 12 F | - |  |
| IQ (mean ± SD) | | 112.59 ± 10.38 | 110.33 ± 10.17 | *p* = 0.46 |  |
| Motion parameters  (mean ± SD) | X (mm)  Y (mm)  Z (mm) | 0.184 ± 0.098  0.314 ± 0.139  0.716 ± 0.482 | 0.225 ± 0.107  0.334 ± 0.198  0.622 ± 0.328 | *p* = 0.16  *p* = 0.69  *p* = 0.43 | |
|  | Pitch (°)  Roll (°)  Yaw (°) | 0.014 ± 0.009  0.006 ± 0.003  0.005 ± 0.003 | 0.013 ± 0.005  0.008 ± 0.004  0.006 ± 0.003 | *p* = 0.62  *p* = 0.08  *p* = 0.47 | |

Notes: F, female; M, male; Sig. - significance in simple analyses of variance (ANOVA

); SD - standard deviation. Motion parameters - six motion parameters (translation: x, y

and z in mm; rotation: pitch, roll and yaw in degrees) were obtained from head

movement correction for each participant.
