# Supplementary material for: Temporal Dynamics and Developmental Maturation of Salience, Default and Central-Executive Network Interactions Revealed by Variational Bayes Hidden Markov Modeling
Source: PLoS Comput Biol. 2016 Dec 13;12(12):e1005138. doi: 10.1371/journal.pcbi.1005138 (PMC5154470; doi:10.1371/journal.pcbi.1005138)
Supplement: S1 File — (DOCX) [file pcbi.1005138.s011.docx]

**Supplementary Materials**

**VB-HMM Model:**

***Prior probabilities***

The prior for initial probability vector $\pi=(\pi_{1},\ldots\ldots,\pi_{K})$ for $K$ states is assumed to be a Dirichlet distribution:

$p\left( \pi\right)=Dir(\pi_{1},\ldots\ldots,\pi_{K}|u_{1}^{\pi},\ldots\ldots,u_{K}^{\pi}$) (S.1)

where the prior parameters for the initial parameters $u_{1}^{\pi},\ldots\ldots,u_{K}^{\pi}$ are each set to initial non-informative values of $\frac{1}{K}$, where the initial number of states $K$ is set to $25.$

Each row of the switching probabilities $A$ is assumed to be an independent Dirichlet distribution:

$$p\left( A \right)=\prod_{k=1}^{K} Dir(A_{k,1},\ldots\ldots,A_{i,K}|u_{i,1}^{A},\ldots\ldots,u_{i,K}^{A}) (S.2)$$

where the initial parameters $u_{i,1}^{A},\ldots\ldots,u_{i,K}^{A}$ are each set to non-informative values of $\frac{1}{K}.$

The observations given the state $k$ is assumed to be a multivariate normal distribution with unknown mean and covariance${\Theta= \{\mu}_{k}, \Sigma_{k}{\}}_{k=1}^{K}$. The prior probability distribution for $\Theta$ is

$$p\left( \Theta\right)=\prod_{k=1}^{K} p\left( \Theta_{k} \right) (S.3)$$

where

$$p\left( \Theta_{k} \right)=NW\left( \mu_{k},\Sigma_{k} \right) (S.4)$$

where $NW(\mu_{k},\Sigma_{k})$ is a normal-Wishart distribution with parameters $a_{k}{,b}_{k}, m_{k},\lambda_{k}$, which are set to non-informative values of $a_{k}=M$ (initial degrees of freedom), ${,b}_{k}={10}^{-3}I_{M}$ (prior sum of squares matrix which is proportional to the inverse covariance or precision matrix)), $m_{k}=0_{M}$ (prior mean), and $\lambda_{k}={10}^{3}$(scale parameter) . Here, $M$ is the number of ROIs, $I_{M}$ is an identity matrix of dimension $M\times M$ and $0_{M}$ is a $M\times1$ vector of zeros.

***Posterior probability distributions for VB-HMM parameters***

The posterior probability for $\pi$ is given by

$q\left( \pi\right)=Dir(\pi_{1},\ldots\ldots,\pi_{K}|W_{1}^{\pi},\ldots\ldots,W_{K}^{\pi}$) (S.5)

$$W_{k}^{\pi}=w_{k}^{\pi}+u_{k}^{\pi} (S.6)$$

where $w_{k}^{\pi}=q(Z_{1}^{s}=k)$, $q(Z_{1}^{s}=k)$ is the posterior probability of the latent state $Z_{1}^{s}$ at $t=1$.

The posterior distribution for switching probabilities $A$ is given by:

$$q\left( A \right)=\prod_{k=1}^{K} Dir(A_{k,1},\ldots\ldots,A_{i,K}|W_{i,1}^{A},\ldots\ldots,W_{i,K}^{A}) (S.7)$$

$$W_{i,j}^{A}=\sum_{s=1}^{S} \sum_{t=1}^{T_{S}-1} w_{i,j}^{s,t}+u_{i,j}^{A} (S.8)$$

where $w_{i,j}^{s,t}$ is the posterior probability of the latent state given by $q(Z_{t}^{s}=i,Z_{t+1}^{s}=j)$.

The posterior distribution for the parameters ${\Theta= \{\mu}_{k}, \Sigma_{k}{\}}_{k=1}^{K}$ is given by:

$$q\left( \Theta\right)=\prod_{k=1}^{K} q\left( \Theta_{k} \right) (S.9)$$

$q\left( \Theta_{k} \right)=NW(\mu_{k},\Sigma_{k})$ with posterior parameters given by:

$$m_{k}^{'}=\frac{\lambda_{k}m_{k}+w_{k}\bar{y}_{k}}{\lambda_{k}+w_{k}} (S.10)$$

$$a_{k}^{'}=a_{k}+w_{k} (S.11)$$

$$S_{k}=\sum_{s=1}^{S} \sum_{t=1}^{T_{s}} w_{k}(y_{t}^{s}-\bar{y}_{k})(y_{t}^{s}-{\bar{y}_{k})}^{T} (S.12)$$

$$b_{k}^{'}={,b}_{k}+S_{k}+\frac{\lambda_{k}w_{k}}{\lambda_{k}+w_{k}}(m_{k}-\bar{y}_{k})(m_{k}-\bar{y}_{k})^{T} (S.13)$$

$$\bar{y}_{k}=\sum_{s=1}^{S} \sum_{t=1}^{T_{S}-1} w_{i,j}^{s,t}y_{t}^{s} (S.14)$$

$$w_{k}=\sum_{s=1}^{S} \sum_{t=1}^{T_{S}-1} w_{k}^{s,t} (S.15)$$

where $m_{k}^{'}$ is the posterior mean, $a_{k}^{'}$, is the posterior degrees of freedom, and $b_{k}^{'}$ is proportional to the posterior inverse covariance for the state $k$. The details of the update equations are given in [[1](#_ENREF_1)]. The posterior distributions for the states $q(Z_{t}^{s}=i)$ and $q(Z_{t}^{s}=i,Z_{t+1}^{s}=j)$ are estimated by the forward and backward algorithm [[1](#_ENREF_1),[2](#_ENREF_2)].

**Validation of VB-HMM**

We validated our VB-HMM model using three simulation models as described below:

1. *Simulation-1*: In this simulation, we created a 2-node model consisting of two distinct states as shown in **Supplementary** **Fig S2a**. The model consists of two states: each “OFF” state is of duration 40 secs wherein the two nodes are negatively correlated with covariance $C_{1}=\left( \begin{matrix} 1 & -1 \\ -1 & 1 \end{matrix} \right)$ and mean of zero; each “ON” state lasts for 20 secs where the nodes are uncorrelated with covariance $C_{2}=\left( \begin{matrix} 1 & 0 \\ 0 & 1 \end{matrix} \right)$ with zero mean. We generate observations simulated from a normal distribution $N(0,C_{1})$ under the “OFF” state and $N(0,C_{2})$ for the “ON” state. Data were generated for five subjects each having a duration of 480 secs.
2. *Simulation-2:* In this model, we use the HMM generative model described in Equations 1-3 to simulate a data set consisting of six nodes. We assume there are two states (**Supplementary** **Fig S3a**) with transition probability $A=\left( \begin{matrix} 0.7723 & 0.2655 \\ 0.2277 & 0.7345 \end{matrix} \right)$and under each state the observations are simulated from a zero-mean multivariate Gaussian distribution with state specific covariance matrices. Five datasets were generated with a duration of 300 secs each.
3. *Simulation-3:* This model consists of two states shown in **Supplementary** **Fig S4a.** In state 1, the first three nodes are correlated, while the other three nodes are uncorrelated with each other; in state 2, the last three nodes are correlated with each other while the first three nodes are uncorrelated. To simulate correlation between the nodes in each state, we take a BOLD fMRI time series of a region from subjects in the Stanford Children cohort 1 and generate time series for each node by adding white Gaussian noise with 20dB noise. The time series for the other three nodes consist of white Gaussian noise with zero mean and standard deviation of 1. Data for five subjects was simulated with each lasting for 232 secs (116 secs for each state).

**Simulation Results**

We applied VB-HMM to uncover the hidden states from the three simulation data sets. For each data set, we initialized the number of underlying states (K) to $25$ and used VB-HMM to discover the optimal number of states.

*Simulation-1*

We applied VB-HMM on all five subject data sets. **Supplementary** **Fig S2** shows the actual states, estimated posterior probabilities and Viterbi path of the states for a representative subject; other subjects have similar results (not shown). Seven states among 25 have non-zero occupancy rates suggesting that VB-HMM penalizes redundant states and discovers the optimal number of states from the data. These are states 16, 24, 20, 2, 18, 10 and 6 (sorted in terms of their occupancy rates) which have occupancy rates of 65.1%, 15.8%, 14.7%, 2.3%, 1.8%, 0.2% and 0.04% respectively. Among these, the top four most dominant states together constitute 98% of the total occupancy rate, with states 24 and 20 well matched to the occupancy rate of the “ON” state and states 16 and 2 well matched to the occupancy rate of the “OFF” state. Interestingly, the estimated Pearson correlation matrices (derived by normalizing the estimated covariance matrices in each state) of these latter two states$, C_{16}=\left( \begin{matrix} 1.0 & -0.9999 \\ -0.9998 & 1.0 \end{matrix} \right)$ and $C_{2}=\left( \begin{matrix} 1.0 & -0.9999 \\ -0.9998 & 1.0 \end{matrix} \right)$, are similar and only differ in high decimal places. These two states have a total combined occupancy rate of 67.4% which is well matched to the occupancy rate of the “OFF” state as well as with the two states’ occurrence with respect to the “OFF” state (**Supplementary** **Fig S2a** and **Supplementary** **Fig S2c)**. In the real fMRI analysis, we merged states having the same community structure. Similarly, states 16 and 2 can therefore be merged together since they represent the same state (“OFF”) in the data.

*Simulation-2*

**Supplementary** **Fig S3** shows the actual states, estimated posterior probabilities and Viterbi path of the states for a representative subject; the other subjects have similar results (not shown). Four states among 25 have non-zero occupancy rates suggesting that VB-HMM penalizes redundant states and discovers the optimal number of states from the data. These are states 20, 8, 14 and 12 which have occupancy rates of 55%, 43%, 1.3%, and 0.7% respectively. Of these, the top two most dominant states together comprise 98% of the total occupancy rate, with state 20 well matched to the occupancy rate of actual state 2 and state 8 well matched with actual state 1.

*Simulation-3*

VB-HMM was applied to all five subject data sets. **Supplementary** **Fig S4** shows the actual states, estimated posterior probabilities and Viterbi path of the states for a representative subject and the other subjects have similar results (not shown). Four states among 25 have non-zero occupancy rates suggesting that VB-HMM penalizes redundant states and discovers the optimal number of states from the data. These are states 5, 15, 2 and 10 which have occupancy rates of 49.6%, 49.6%, 0.04% and 0.04% respectively. Of these four, the top two most dominant states constitute 99.2% of the total occupancy rate, with state 5 well matched to the occupancy rate of actual state 1 and state 15 well matched with actual state 2.

**Null model of intrinsic brain dynamics**

We examined state properties including occupancy rate, mean lifetime, and transition probabilities in a null model. The null model was constructed using 100 surrogate datasets; each surrogate dataset was obtained by applying a Fourier transform to the observed signal in each region. We randomized the phase by adding a random phase shift sampled in the interval [0, 2π]. Shifts at the same frequency from different time series and at different frequencies were all chosen independently. An inverse Fourier transform was then applied to generate an instance of surrogate data. Randomization of the phase response creates a stationary stochastic time series with the same auto-correlation structure as the original data but without non-stationarities or cross correlations. We then repeated our entire analysis on the surrogate data generated by the null model. Null model was created for each of the four cohorts – HCP Cohort 1, HCP Cohort 2, Stanford Adult Cohort, Stanford Child Cohort.

Results of these analyses revealed the following: (1) As expected, VB-HMM uncovered on an average 1-2 states in the surrogate datasets, (2) the mean occupancy rate was 4%, (3) the mean lifetime was high (34.32 s). This could be attributed to the existence of one-two states in the surrogate datasets, (4) the mean self state-transition probability was 0.99-1 while the cross state transition probability was 0-0.01, and (5) The mean occupancy rates, mean lifetime and state transition probability of the two dominant states that were the focus of our investigation were significantly different from the corresponding values observed in the surrogate datasets. These results were observed for HCP cohort 1 and HCP cohort 2. For surrogate datasets generated from Stanford Adults and Stanford Children fMRI data, we observed similar results except for the mean lifetime which was 18.56s.

**References**

1. Ji SH, Krishnapuram B, Carin L (2006) Variational Bayes for continuous hidden Markov models and its application to active learning. Ieee Transactions on Pattern Analysis and Machine Intelligence 28: 522-532.

2. Bishop C (2006) Pattern Recognition and Machine Learning: Springer.
